# Determination of globotriaosylceramide analogs in the organs of a mouse model of Fabry disease

**DOI:** 10.1074/jbc.RA120.012665

**Published:** 2020-03-16

**Authors:** Satoshi Ishii, Atsumi Taguchi, Nozomu Okino, Makoto Ito, Hiroki Maruyama

**Affiliations:** ‡ Department of Matrix Medicine, Faculty of Medicine, Oita University, Oita 879-5593, Japan; § Biochemical Laboratory, GlycoPharma Corporation, Oita 870-0822, Japan; ¶ Department of Clinical Nephroscience, Niigata University Graduate School of Medical and Dental Sciences, Niigata 951-8510, Japan; ‖ Department of Bioscience and Biotechnology, Graduate School of Bioresource and Bioenvironmental Sciences, Kyushu University, Fukuoka 819-0395, Japan

**Keywords:** sphingolipid, ceramide, genetic disease, lysosomal storage disease, mouse, Fabry disease, globotriaosylceramide, globotriaosylceramide analogs, N-deacylase, GlatmTg(CAG-A4GALT), Fabry mouse model, alpha-galactosidase A, X-linked disease, lipid disorder, glycosphingolipid, lysosomal storage disorder

## Abstract

Fabry disease is a heritable lipid disorder caused by the low activity of α-galactosidase A and characterized by the systemic accumulation of globotriaosylceramide (Gb3). Recent studies have reported a structural heterogeneity of Gb3 in Fabry disease, including Gb3 isoforms with different fatty acids and Gb3 analogs with modifications on the sphingosine moiety. However, Gb3 assays are often performed only on the selected Gb3 isoforms. To precisely determine the total Gb3 concentration, here we established two methods for determining both Gb3 isoforms and analogs. One was the deacylation method, involving Gb3 treatment with sphingolipid ceramide *N*-deacylase, followed by an assay of the deacylated products, globotriaosylsphingosine (lyso-Gb3) and its analogs, by ultra-performance LC coupled to tandem MS (UPLC-MS/MS). The other method was a direct assay established in the present study for 37 Gb3 isoforms and analogs/isoforms by UPLC-MS/MS. Gb3s from the organs of symptomatic animals of a Fabry disease mouse model were mainly Gb3 isoforms and two Gb3 analogs, such as Gb3(+18) containing the lyso-Gb3(+18) moiety and Gb3(−2) containing the lyso-Gb3(−2) moiety. The total concentrations and Gb3 analog distributions determined by the two methods were comparable. Gb3(+18) levels were high in the kidneys (24% of total Gb3) and the liver (13%), and we observed Gb3(−2) in the heart (10%) and the kidneys (5%). These results indicate organ-specific expression of Gb3 analogs, insights that may lead to a deeper understanding of the pathophysiology of Fabry disease.

## Introduction

Fabry disease (OMIM 301500) is an X-linked inherited disorder caused by the accumulation of glycosphingolipids, predominantly globotriaosylceramide (Gb3),^2^ in organs throughout the body and results from deficiencies in the activity of a lysosomal hydrolase called α-galactosidase A (EC 3.2.1.22) (1). Patients with Fabry disease present various clinical symptoms and manifestations, including peripheral neuropathy, stroke, gastrointestinal disorders, renal impairment, and cardiovascular disease, leading to premature death (2, 3).

For the study of Fabry disease, α-galactosidase A (*Gla*) knockout mouse lines were generated in two different laboratories; however, neither displayed any abnormalities throughout its lifespan (4, 5). We previously cross-bred asymptomatic *Gla* knockout (C57BL/6J;129S4-*Gla^tm1kul^*) mice with transgenic mice expressing human α1,4-galactosyltransferase (A4GALT), a Gb3 synthase, and generated a symptomatic *Gla^tm^Tg(CAG-A4GALT)* Fabry mouse model with renal impairment (6, 7). Using this mouse model, we demonstrated that increased Gb3 accumulation caused renal impairment, but the pathophysiology of Fabry disease remains to be clarified.

Several treatment strategies for Fabry disease have been developed, and enzyme replacement therapy (ERT) and pharmacological chaperone therapy have been available since 2001 (8, 9) and 2016 (10), respectively. Substrate reduction therapy, the subject of an ongoing phase 3 clinical trial, is another promising treatment option (11). As treatment options increase, the need for biomarkers to select suitable treatments for individual patients and evaluate therapeutic effects has increased rapidly.

Gb3 has been used as a biomarker to determine ERT efficacy (12, 13). In 2008, Aerts *et al.* (14) introduced another biomarker, globotriaosylsphingosine (lyso-Gb3), which is present at high levels in the plasma of patients with Fabry disease and shows good responses to ERT (15). Recent metabolomic studies have described the presence of eight different lyso-Gb3 analogs with sphingosine moiety modifications in the plasma and urine of patients with Fabry disease (16, 17). Metabolomic studies focusing on the Gb3 structure (18, 19) also identified Gb3 isoforms (Gb3 containing various fatty acids without sphingosine modifications) and Gb3 analogs (Gb3 with various sphingosine modifications) in the plasma and urine of patients with Fabry disease. These variations in sphingosine and fatty acid moieties contribute to the structural heterogeneity of Gb3.

Several assays for Gb3 determination have been described previously, such as HPLC of benzoyl-derivatives (20), TLC-orcinol staining (21), and Shiga toxin B subunit–binding assays (22, 23); however, recently, Gb3 analysis was performed by LC coupled to tandem MS (LC-MS/MS) because of its high specificity and sensitivity (13). Gb3 concentrations in the plasma of patients with Fabry disease are often assayed by measuring the 10 most abundant Gb3 isoforms (13, 24); however, Manwaring *et al.* (19) described the presence of >24 Gb3 isoforms/analogs in the plasma of these patients. The percentages of these Gb3 analogs could not be measured because of the presence of structural isomers. For example, Gb3(d18:1)(C16:1), Gb3(d18:1)(C18:1), Gb3(d18:1)(C20:1), Gb3(d18:1)(C22:1), and Gb3(d18:1)(C24:1) cannot be distinguished from Gb3(d18:2)(C16:0), Gb3(d18:2)(C18:0), Gb3(d18:2)(C20:0), Gb3(d18:2)(C22:0), and Gb3(d18:2)(C24:0), respectively.

**Figure 1.**
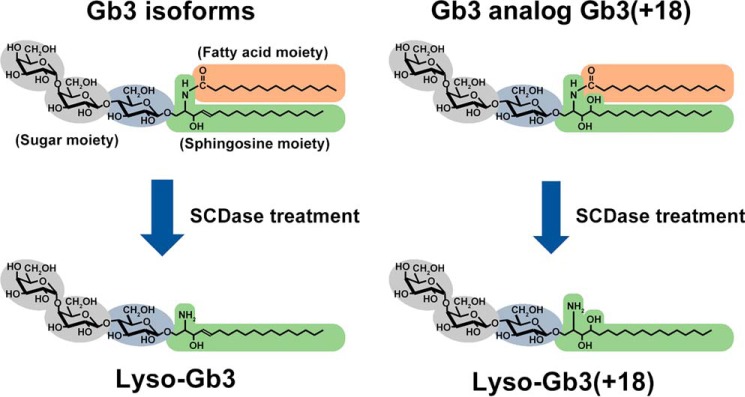
**Schematic representation of SCDase digestion of Gb3 isoforms and analog/isoforms.** The structure of lyso-Gb3(+18) is putative and based on its mass data. The heterogeneity of Gb3 isoforms caused by various fatty acids is simplified by SCDase treatment.

The purpose of this study was to establish analytical methods to determine the total Gb3 concentration and the distribution of Gb3 analogs. Because *N*-acylated fatty acids contribute to Gb3 heterogeneity, we removed the fatty acids from Gb3 with sphingolipid ceramide *N*-deacylase (SCDase (25); Fig. 1), which has previously been used for the deacylation of gangliosides (26). Then we identified deacylated products (lyso-Gb3 and its analogs) using ultra-performance (UP)LC-MS/MS (27), enabling the determination of all types of Gb3 isoforms and analogs. We designated Gb3 analogs containing the lyso-Gb3(−2) and lyso-Gb3(+18) moieties as Gb3(−2) and Gb3(+18), respectively. In our assay, the structural isomers mentioned above were divided into two groups: Gb3 isoforms (Gb3(d18:1)(C16:1), Gb3(d18:1)(C18:1), etc.) determined by lyso-Gb3 and Gb3(−2) analog/isoforms (Gb3(d18:2)(C16:0), Gb3(d18:2)(C18:0), etc.) measured by lyso-Gb3(−2). To confirm that the Gb3 content was accurately determined by the Gb3 deacylation assay, in this study, we also developed a Gb3 direct assay via 37 synthesized Gb3 molecules and investigated the organ-specific expression of Gb3 molecules in symptomatic Fabry model mice.

## Results and discussion

### Optimal conditions for Gb3 deacylation with SCDase

To determine the optimal amount of enzyme and incubation time required, standard Gb3 was incubated with SCDase at various concentrations and for different lengths of time (Fig. S1). Dose- and time-dependent increases in lyso-Gb3 were observed after incubating Gb3 with SCDase, and lyso-Gb3 production reached a plateau at SCDase concentrations of >4 μg/ml and at incubation periods of >30 min. The data suggested that Gb3 is a good substrate for SCDase and that constant Gb3 deacylation can be achieved through incubation with 5 μg/ml SCDase for 1 h. Almost all of the deacylated products from standard Gb3 were lyso-Gb3, and good linearity was observed between the Gb3 concentration at 0.6–20 μg/ml and the amount of lyso-Gb3 produced.

### Deacylation of Gb3 from different organs of Gla^tm^Tg(CAG-A4GALT) mice

Next, we assessed whether assaying Gb3 after SCDase deacylation was possible with heterogeneous Gb3 containing various isoforms and analogs. As different Gb3 isoforms and analogs have been detected in different mouse organs (28, 29), we purified Gb3 from the heart, kidneys, spleen, and liver from *Gla^tm^Tg(CAG-A4GALT)* mice. The total Gb3 purified from each organ was weighed, and 4 μg of each preparation was subjected to TLC and visualized with orcinol-sulfuric acid reagent, as described previously (30). The Gb3 preparations from the heart and spleen were observed as almost single bands, and the Gb3 preparations from the kidneys and liver showed more than three bands, which may contain various polar Gb3 types (Fig. S2). Purified Gb3 (10 μg/ml) from each organ was digested with SCDase, and the deacylated products were analyzed by UPLC-MS/MS. The peak areas of lyso-Gb3 and its analogs are presented in Table 1, compared with those of standard Gb3. The highest peak area was observed for lyso-Gb3 in all preparations, and obvious lyso-Gb3(−2) and lyso-Gb3(+18) peaks were detected as deacylated products of Gb3 purified from mouse organs. The percentages of Gb3(−2) were 10.3, 5.1, 1.9, and 2.6% in the heart, kidneys, spleen, and liver, respectively. The percentages of Gb3(+18) were 1.7, 24.6, 1.1, and 13.3% in the heart, kidneys, spleen, and liver, respectively. Although the pattern of deacylated Gb3 differed from that of standard Gb3, the total peak area of lyso-Gb3 and its analogs was identical in all purified Gb3 preparations. This result indicates that constant digestion by SCDase occurred in both Gb3 isoforms and Gb3 analogs. The presence of Gb3 analogs containing lyso-Gb3 analogs has been suggested in *Gla* knockout mouse organs (28, 29) and the plasma of patients with Fabry disease (19), but this is the first experimental data demonstrating the presence of Gb3 analogs in mouse organs. LC-MS/MS has been widely used to determine Gb3 content in the plasma of patients with Fabry disease (13) and in mouse organs (31, 32), and it can detect selected Gb3 isoforms but not Gb3 analogs. In this study, Gb3 analogs constituted <2% of the standard Gb3 from Matreya LLC; however, significant amounts of Gb3 analogs were observed in the heart, kidneys, and liver (∼12, 30, and 16%, respectively) of our mouse model (Table 1).

**Table 1:** **Peak areas of lyso-Gb3 and its analogs prepared from a 10 μg/ml Gb3 solution after SCDase digestion**

Purified Gb3	Peak area of deacylated Gb3 determined by UPLC-MS/MS									Total area ± S.D.
	Lyso-Gb3	Lyso-Gb3 analogs								
		(−28)	(−12)	(−2)	(+14)	(+16)	(+18)	(+34)	(+50)	
Standard*^a^*	5677	1	0	19	25	0	21	0	0	5742 ± 619
Heart*^b^*	4828	9	0	572	3	0	97	0	0	5509 ± 415
Kidney	3795	0	0	277	2	0	1330	0	0	5403 ± 205
Spleen	5522	0	0	110	1	0	67	1	0	5701 ± 231
Liver	4919	10	0	155	3	0	785	2	0	5874 ± 376

*^a^* Standard Gb3 is Gb3 from porcine RBC.

*^b^* Heart, kidney, spleen, and liver Gb3 were purified from the organs of *Gla^tm^Tg(CAG-A4GALT)* mice. Results are the mean of four independent assays.

**Figure 2.**
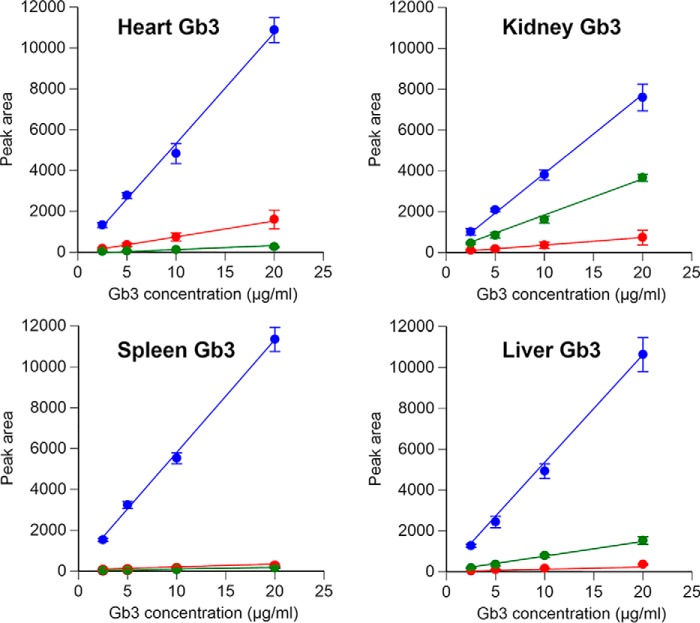
**Calibration curves of purified Gb3 from the organs of *Gla^tm^Tg(CAG-A4GALT)* mice.** Gb3 preparations purified from mouse organs were treated with SCDase, and the deacylated products, lyso-Gb3 (*blue*), lyso-Gb3(−2) (*red*), and lyso-Gb3(+18) (*green*), were extracted and assayed by UPLC-MS/MS. Results are the mean ± S.D. (*error bars*) of four independent assays.

Next, Gb3 calibration curves for the four purified Gb3 preparations from 2.5 to 20 μg/ml were determined (Fig. 2). Good linearity was observed between the peak area of lyso-Gb3 and its analogs and the concentration of all four Gb3 preparations. These results suggest that the SCDase deacylation method is applicable to various Gb3 isoforms and analogs.

### Properties of purified lyso-Gb3 and its analogs

Next, the deacylated products were purified to clarify their characterization. Purified kidney Gb3 was digested with SCDase, and the deacylated products were separated by HPLC. The final preparations of purified lyso-Gb3, lyso-Gb3(−2), and lyso-Gb3(+18) were designated as LG3, LG3(−2), and LG3(+18), respectively. At adjusted concentrations of 1 μg/ml, the preparations of LG3, LG3(−2), and LG3(+18) displayed similar peak areas for each analog in the UPLC-MS/MS conditions, and the purities were 98, 99, and 98% for lyso-Gb3, lyso-Gb3(−2), and lyso-Gb3(+18), respectively (Table 2). LG3, LG3(−2), and LG3(+18) displayed single precursor ion mass spectra at 786.32, 784.32, and 804.38 *m*/*z*, respectively, and single product ion mass spectra at 282.23, 280.24, and 318.26 *m*/*z*, respectively (Fig. S3).

**Table 2:** **Characterization of purified lyso-Gb3 and its analogs** The concentrations of lyso-Gb3, lyso-Gb3(−2), and lyso-Gb3(+18) in final samples at 1 μg/ml were determined by UPLC-MS/MS. Results are the mean of three independent assays.

Samples	Peak area determined by UPLC-MS/MS		
	Lyso-Gb3	Lyso-Gb3(−2)	Lyso-Gb3(+18)
LG3	154,200	8	3460
LG3(−2)	1536	138,500	17
LG3(+18)	3526	1	159,600

### Synthesis of Gb3 isoforms and analogs/isoforms

**Figure 3.**
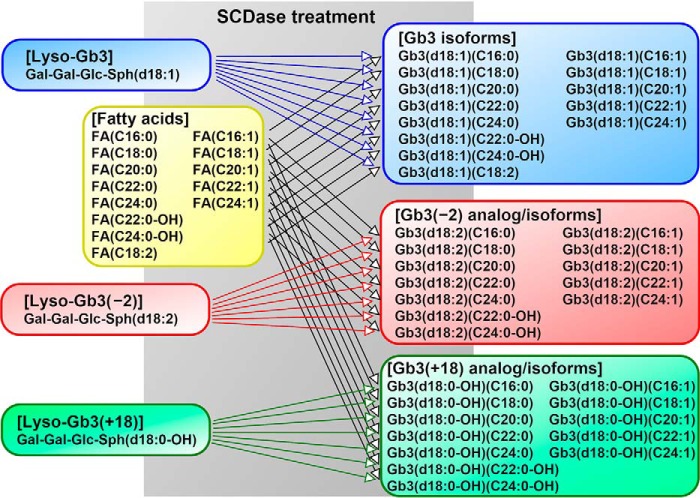
**Synthesis of Gb3 isoforms and analog/isoforms by treatment with SCDase.** Gb3 molecules were synthesized from purified lyso-Gb3 and its analogs and commercially available fatty acids. *Gal*, galactose; *Glc*, glucose; *Sph*, sphingoid base; *FA*, fatty acid.

To establish the Gb3 direct assay conditions, 13 Gb3 isoforms, 12 Gb3(−2) analog/isoforms, and 12 Gb3(+18) analog/isoforms were synthesized by the reverse reaction of SCDase from LG3, LG3(−2), and LG3(+18), respectively, as well as 13 commercially available fatty acids, including saturated, nonsaturated, and hydroxy fatty acids (Fig. 3). The cross-contamination of each Gb3 molecule was also investigated. The preparation of the synthesized Gb3 isoform containing stearic acid was designated as LG3/C18:0, and Gb3 analog preparations with stearic acid were designated as LG3(−2)/C18:0 or LG3(+18)/C18:0.

### Mass detection of synthesized Gb3 containing C18 fatty acid

**Figure 4.**
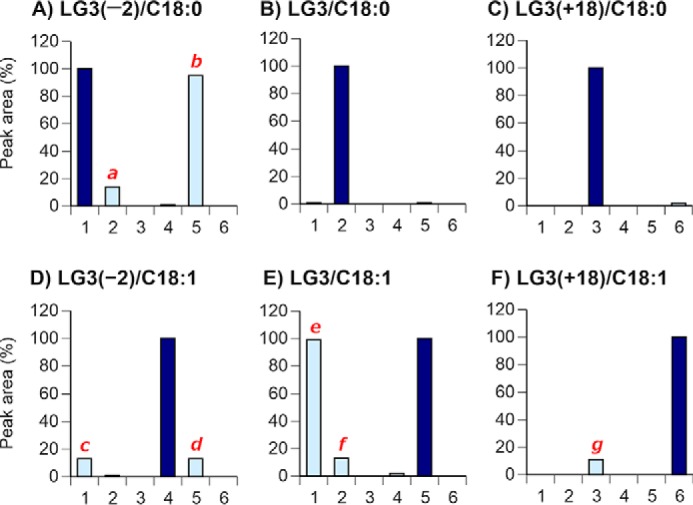
**Synthesized Gb3 isoforms and analogs with C18 fatty acids.** Each synthesized Gb3 preparation was applied to UPLC-MS/MS, and six different transition parameters were determined at a narrow retention time range. *Numbers* along the *x* axis are transition parameters: *1*, 1072.7 > 910.6 for Gb3(d18:2)(C18:0); *2*, 1074.7 > 912.6 for Gb3(d18:1)(C18:0); *3*, 1092.7 > 930.6 for Gb3(d18:0-OH)(C18:0); *4*, 1070.7 > 908.6 for Gb3(d18:2)(C18:1); *5*, 1072.7 > 910.6 for Gb3(d18:1)(C18:1); *6*, 1090.7 > 928.6 for Gb3(d18:0-OH)(C18:1). Retention times are 1.61 ± 0.04 min (*A*), 1.69 ± 0.04 min (*B*), 1.65 ± 0.04 min (*C*), 1.54 ± 0.04 min (*D*), 1.61 ± 0.04 min (*E*), and 1.57 ± 0.04 min (*F*), respectively. Peaks other than the authentic peaks are designated as peaks *a* to *g*. The authentic peak of the molecule (*dark blue bar*) was defined as 100%. Results are the mean of three independent assays.

To establish a selective assay for each Gb3 molecule, the Gb3 isoforms and analogs/isoforms containing C18 fatty acids were first analyzed in different mass conditions (Fig. 4). For example, LG3(−2)/C18:0 (equal to Gb3(d18:2)(C18:0)) was recovered at a retention time of 1.61 ± 0.04 min when it was applied to the UPLC column, as described in Table 3, and it was analyzed by six different transition parameters: parameter 1 (1072.7 > 910.6, Gb3(d18:2)(C18:0)), parameter 2 (1074.7 > 912.6, Gb3(d18:1)(C18:0)), parameter 3 (1092.7 > 930.6, Gb3(d18:0-OH)(C18:0)), parameter 4 (1070.7 > 908.6, Gb3(d18:2)(C18:1)), parameter 5 (1072.7 > 910.6, Gb3(d18:1)(C18:1)), and parameter 6 (1090.7 > 928.6, Gb3(d18:0-OH)(C18:1)). As shown in Fig. 4*A*, the highest peak was observed at parameters 1 and 5 (peak *b*) because they are structural isomers and have the same transition parameter. The small peak *a* (14% of the highest) was detected by parameter 2. The small peaks *c* (13%), *d* (13%), *f* (13%), and *g* (11%) are also observed in Fig. 4 (*D–F*). All small peaks were detected at the transition parameter plus 2 atomic mass units of the transition parameter for the authentic Gb3 molecule. Based on these data, we suggest that a double bond of sphingoid base or fatty acid moieties may be saturated while ionization occurs in the assay system to some extent. These properties were also observed in other Gb3 molecules with different chain length fatty acids (see Figs. S4–S7).

**Table 3:** **Synthesized Gb3 isoforms and analogs/isoforms and their basic mass conditions and retention time**

Compound number	Gb3 molecules	Transition	Retention time
			*min*
	Gb3(d18:2) (C17:0)*^a^*	1058.7 > 896.6	1.56 ± 0.04
1	Gb3(d18:2) (C16:1)	1042.7 > 880.6	1.42 ± 0.04
2	Gb3(d18:2) (C16:0)	1044.7 > 882.6	1.53 ± 0.04
3	Gb3(d18:1) (C16:1)	1044.7 > 882.6	1.53 ± 0.04
4	Gb3(d18:1) (C16:0)	1046.7 > 884.6	1.61 ± 0.04
5	Gb3(d18:0-OH) (C16:1)	1062.7 > 900.6	1.47 ± 0.04
6	Gb3(d18:0-OH) (C16:0)	1064.7 > 902.6	1.57 ± 0.04
7	Gb3(d18:2) (C18:1)	1070.7 > 908.6	1.54 ± 0.04
7	Gb3(d18:1) (C18:2)	1070.7 > 908.6	1.54 ± 0.04
8	Gb3(d18:2) (C18:0)	1072.7 > 910.6	1.61 ± 0.04
9	Gb3(d18:1) (C18:1)	1072.7 > 910.6	1.61 ± 0.04
10	Gb3(d18:1) (C18:0)	1074.7 > 912.6	1.69 ± 0.04
11	Gb3(d18:0-OH) (C18:1)	1090.7 > 928.6	1.57 ± 0.04
12	Gb3(d18:0-OH) (C18:0)	1092.7 > 930.6	1.65 ± 0.04
13	Gb3(d18:2) (C20:1)	1098.7 > 936.6	1.62 ± 0.04
14	Gb3(d18:2) (C20:0)	1100.7 > 938.6	1.69 ± 0.04
15	Gb3(d18:1) (C20:1)	1100.7 > 938.6	1.69 ± 0.04
16	Gb3(d18:1) (C20:0)	1102.7 > 940.6	1.77 ± 0.04
17	Gb3(d18:0-OH) (C20:1)	1118.7 > 956.6	1.65 ± 0.04
18	Gb3(d18:0-OH) (C20:0)	1120.7 > 958.6	1.73 ± 0.04
19	Gb3(d18:2) (C22:1)	1126.7 > 964.6	1.66 ± 0.04
20	Gb3(d18:2) (C22:0)	1128.7 > 966.6	1.76 ± 0.04
21	Gb3(d18:1) (C22:1)	1128.7 > 966.6	1.76 ± 0.04
22	Gb3(d18:1) (C22:0)	1130.7 > 968.6	1.84 ± 0.04
23	Gb3(d18:0-OH) (C22:1)	1146.7 > 984.6	1.72 ± 0.04
24	Gb3(d18:0-OH) (C22:0)	1148.7 > 986.6	1.81 ± 0.04
25	Gb3(d18:2) (C24:1)	1154.7 > 992.6	1.75 ± 0.04
26	Gb3(d18:1) (C24:2)*^b^*	1154.7 > 992.6	1.75 ± 0.04
27	Gb3(d18:2) (C24:0)	1156.7 > 994.6	1.83 ± 0.04
28	Gb3(d18:1) (C24:1)	1156.7 > 994.6	1.83 ± 0.04
29	Gb3(d18:1) (C24:0)	1158.7 > 996.6	1.91 ± 0.04
30	Gb3(d18:0-OH) (C24:1)	1174.7 > 1012.6	1.79 ± 0.04
31	Gb3(d18:0-OH) (C24:0)	1176.7 > 1014.6	1.88 ± 0.04
32	Gb3(d18:2) (C22:0-OH)	1144.7 > 982.6	1.75 ± 0.04
33	Gb3(d18:2) (C24:0-OH)	1172.7 > 1010.6	1.81 ± 0.04
34	Gb3(d18:1) (C22:0-OH)	1146.7 > 984.6	1.83 ± 0.04
35	Gb3(d18:1) (C24:0-OH)	1174.7 > 1012.6	1.90 ± 0.04
36	Gb3(d18:0-OH) (C22:0-OH)	1164.7 > 1002.6	1.80 ± 0.04
37	Gb3(d18:0-OH) (C24:0-OH)	1192.7 > 1030.6	1.87 ± 0.04

*^a^* Internal standard.

*^b^* Gb3(d18:1)(C24:2) was not synthesized, and its retention time was predicted from the data of Gb3(d18:2)(C24:1).

### Cross-contamination of Gb3 mass data with similar structures

**Figure 5.**
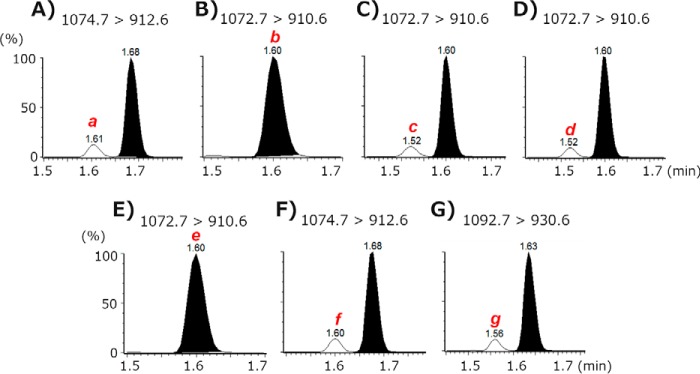
**UPLC-MS/MS chromatograms of the mixed Gb3 preparations containing C18 fatty acids.** *A*, LG3(−2)/C18:0 and LG3/C18:0 were mixed and applied to UPLC-MS/MS and detected with the transition parameter 2 in the legend to Fig. 4 (1074.7 > 912.6) at the retention time 1.69 ± 0.04 min. *B*, LG3(−2)/C18:0 and LG3/C18:1 mixture, parameter 5 (1072.7 > 910.6), at 1.61 ± 0.04 min. *C*, LG3(−2)/C18:1 and LG3(−2)/C18:0 mixture, parameter 1 (1072.7 > 910.6), at 1.61 ± 0.04 min. *D*, LG3(−2)/C18:1 and LG3/C18:1 mixture, parameter 5 (1072.7 > 910.6), at 1.61 ± 0.04 min. *E*, LG3/C18:1 and LG3(−2)/C18:0 mixture, parameter 1 (1072.7 > 910.6), at 1.61 ± 0.04 min. *F*, LG3/C18:1 and LG3/C18:0 mixture, parameter 2 (1074.7 > 912.6), at 1.69 ± 0.04 min. *G*, LG3(+18)/C18:1 and LG3(+18)/C18:0 mixture, parameter 3 (1092.7 > 930.6), at 1.65 ± 0.04 min. Peaks *a* to *g* are corresponded with peaks *a* to *g* in Fig. 4.

Next, we assessed whether peaks other than the authentic peak observed in Fig. 4 can be separated from an authentic Gb3 peak under our UPLC conditions by mixing the same amount of two synthesized Gb3 preparations; for example, LG3(−2)/C18:0 and LG3/C18:0 were mixed and assayed for peak *a*. As shown in Fig. 5*A*, peak *a* of LG3(−2)/C18:0 was completely separated from the authentic peak of LG3/C18:0. In the same manner, other small peaks *c*, *d*, *f*, and *g* were also separated from authentic peaks of LG3(−2)/C18:0, LG3/C18:1, LG3/C18:0, and LG3(+18)/C18:0, respectively (Fig. 5, *C*, *D*, *F*, and *G*). However, structural isomers LG3(−2)/C18:0 and LG3/C18:1 were recovered in the same retention time, and peaks *b* and *e* could not be separated under the UPLC conditions in this study. These data were the same for other Gb3 preparations with different fatty acids; all small peaks did not affect any Gb3 assay in the study conditions (see Figs. S8–S11).

### Separation of structural isomers

**Figure 6.**
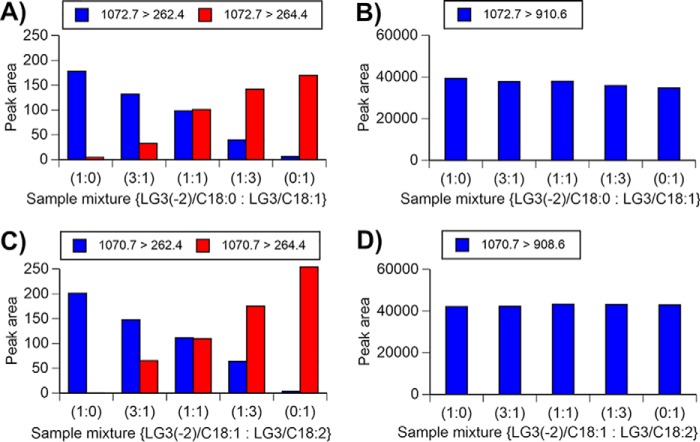
**Detection of structural isomers of Gb3 with C18 fatty acids.** The mixtures of LG3(−2)/C18:0 and LG3/C18:1 at the indicated ratio were determined by the backbone mass conditions (*A*) or basic mass condition (*B*). The mixtures of LG3(−2)/C18:1 and LG3/C18:2 were also subjected to the backbone assay (*C*) and basic assay (*D*). Results are the mean of three independent assays.

Shaner *et al.* (33) reported that sphingolipid subspecies can be distinguished by the production of a *m*/*z* 264.4 fragment, which is a sphingosine (d18:1) base backbone. Therefore, the most effective mass conditions for the determination of sphingosine and sphingadiene (d18:2) backbones were investigated in this study. The most effective conditions for cone voltage and collision energy were determined, resulting in them being set at 165 and 85 V, respectively, which showed the highest MRM of LG3(−2)/C18:0 and LG3/C18:1 at 1072.7 > 262.4 and 1072.7 > 264.4, respectively. To verify their selective determinations, mixtures of LG3(−2)/C18:0 and LG3/C18:1 at different ratios were prepared and assayed via backbone mass conditions (1072.7 > 262.4 and 1072.7 > 264.4) and basic mass conditions (1072 > 910.6). The peak area detected by the backbone mass conditions corresponded positively to their mixing ratio (Fig. 6*A*), although its sensitivity was much lower than that in basic mass conditions (Fig. 6*B*). From these data, a determination strategy for the content of Gb3(d18:2)(C18:0) and Gb3(d18:1)(C18:1) was established as follows. The total content of the structural isomers was assayed via basic mass conditions, and the individual contents were calculated based on their ratio determined by their backbone mass conditions. This method is applicable for higher unsaturated Gb3 molecules Gb3(d18:2)(C18:1) and Gb3(d18:1)(C18:2) (Fig. 6, *C* and *D*) and Gb3 structural isomers with other chain-length fatty acids (see Fig. S12).

### Assay conditions for Gb3 containing hydroxy fatty acids

Gb3 isoforms and analogs containing 2-hydroxy C22:0 and 2-hydroxy C24:0 fatty acids were prepared using the same method as that for Gb3 containing saturated and unsaturated fatty acids. The cross-contamination of each molecule on the mass data was determined between Gb3 molecules containing the same chain-length fatty acids (Fig. S13). LG3(−2)/C22:0-OH showed the highest peak area at the authentic acquisition parameter (1144.7 > 982.6, Gb3(d18:2)(C22:0-OH)) and minor peaks *a* (16%) and *b* (17%) at acquisition parameters (1146.7 > 984.6, Gb3(d18:0-OH)(C22:1)) and (1146.7 > 984.6, Gb3(d18:1)(C22:0-OH)), respectively, which were plus 2 atomic mass units of the authentic acquisition parameter. LG3/C22:0-OH (1146.7 > 984.6, Gb3(d18:1)(C22:0-OH)) also showed a minor peak *c* (1148.7 > 986.6, Gb3(d18:0-OH)(C22:0)) and major peak *d* (1146.7 > 984.6, Gb3(d18:0-OH)(C22:1)). These properties were identical in Gb3 molecules with 2-hydroxy C24:0 fatty acid.

Next, the cross-contamination of these eight peaks on the mass data of the authentic Gb3 molecule in our assay method was assessed by mixing the same amount of two synthesized Gb3 molecules and assaying them (Fig. S14). Although peaks *b*, *d*, *f*, and *h* were clearly distinguished from the peaks of the authentic Gb3 molecules, peaks *a*, *c*, *e*, and *g* were ingested into the peak of the authentic Gb3 molecules and slightly increased their mass data (see Fig. S15). Therefore, the peak areas of Gb3(d18:0-OH)(C22:1), Gb3(d18:0-OH)(C22:0), Gb3(d18:0-OH)(C24:1), and Gb3(d18:0-OH)(C24:0) should be modified based on the contents of Gb3(d18:2)(C22:0-OH), Gb3(d18:1)(C22:0-OH), Gb3(d18:2)(C24:0-OH), and Gb3(d18:1)(C24:0-OH), respectively.

### Application for animal study

**Figure 7.**
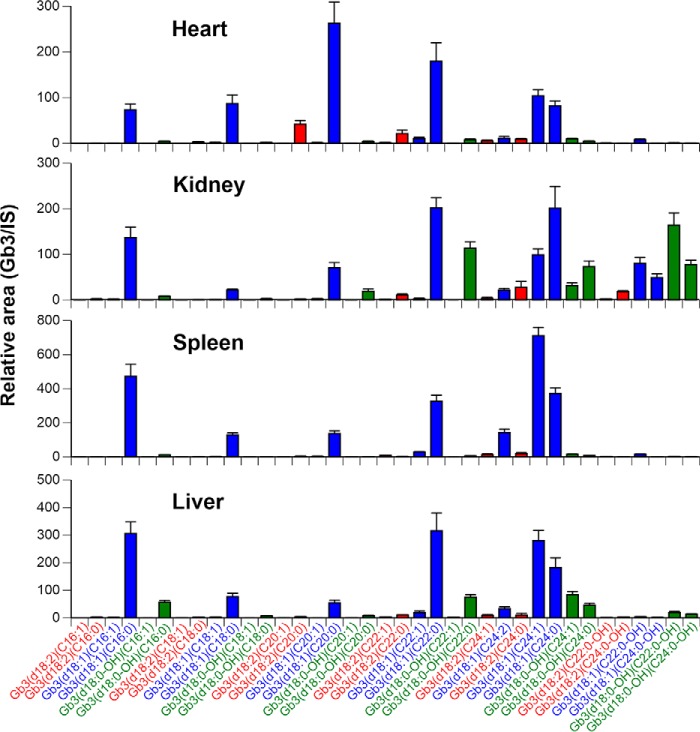
**Distributions of Gb3 isoforms and analogs/isoforms in organs from 20-week-old *Gla^tm^Tg(CAG-A4GALT)* mice.** Results are the mean ± S.D. (*error bars*) of four homogenates (1 mg of protein/ml) from mouse organs. *Blue bar*, Gb3 isoforms; *red bar*, Gb3(−2) analog/isoforms; *green bar*, Gb3(+18) analog/isoforms.

From the above experiments that synthesized 37 Gb3 isoforms and analogs, a Gb3 direct assay was established, and four major organs from 20-week-old *Gla^tm^Tg(CAG-A4GALT)* mice (male, *n* = 4) were utilized (Fig. 7). A symptomatic *Gla^tm^Tg(CAG-A4GALT)* Fabry mouse model generated in our previous study (6) showed renal impairment caused by the progressive accumulation of Gb3. Medullary thick ascending limb dysfunction, an inability to concentrate urine, induced water- and salt-loss phenotypes observed in *Gla^tm^Tg(CAG-A4GALT)* mice by 10 weeks of age (7). Six major Gb3 isoforms (Gb3(d18:1)(C16:0), Gb3(d18:1)(C18:0), Gb3(d18:1)(C20:0), Gb3(d18:1)(C22:0), Gb3(d18:1)(C24:1), and Gb3(d18:1)(C24:0)) were observed in all organs with some varieties of the ratio; for example, Gb3(d18:1)(C20:0) was the highest in the heart but relatively low in other organs. The relatively high contents of Gb3(−2) analog/isoforms in the heart and kidneys were observed, and it was further elucidated that the fatty acids of Gb3(−2) analog/isoforms were distinguishable between the heart and kidneys; in other words, Gb3(d18:2)(C20:0) and Gb3(d18:2)(C22:0) were at high levels in the heart, whereas Gb3(d18:2)(C24:0) and Gb3(d18:2)(C24:0-OH) were at high levels in the kidneys. A relatively high amount of hydroxylated Gb3 (Gb3 containing hydroxylated fatty acid and/or hydroxylated sphingosine moiety, Gb3(+18)) was observed in the kidneys from our symptomatic *Gla^tm^Tg(CAG-A4GALT)* mice. The decrease in Gb3 accumulation in the heart and kidneys is one of the therapeutic targets for Fabry disease. The organ-specific Gb3 may be useful as a monitoring biomarker.

### Comparison between enzymatic deacylation and direct assays

Next, the total Gb3 concentration and distribution of Gb3 analogs in mouse organs between two methods were established in the present study (Table 4). The data on four organs were comparable between two methods; however, the total Gb3 contents in the kidneys and spleen determined by direct assay were slightly lower than those determined by deacylation assay. The percentage of Gb3 isoform in all four organs was lower in the direct assay, and the percentage of Gb3(+18) was higher instead. These data indicate that some Gb3 isoforms in mouse organs may be missed when investigating using direct assays. Although 37 Gb3 molecules were synthesized with the commercially available fatty acids, there is a possibility that mouse organs contained Gb3 isoforms with fatty acids which have different number of double bonds and/or carbon chain lengths. Total Gb3 content and Gb3-analog distributions may be more precisely determined via a deacylation assay. To compare with the current LC-MS/MS method, we also determined total Gb3 by the 10 most abundant Gb3 isoforms (76.2 ± 13.1, 53.7 ± 7.1, 124.2 ± 11.6, and 116.9 ± 18.9 μg/mg protein in the heart, kidneys, spleen, and liver, respectively). They were markedly lower than those by our present method in Table 4. These data indicate that we must measure the Gb3 analog contents for the precise Gb3 determination. Although we determined the molecular structure of Gb3(+18), it may contain phytosphingosine, which is produced in mammalian kidneys (34, 35). The organ-specific distribution of Gb3 analogs/isoforms must be caused by the difference of ceramide synthesis, such as sphingosine modification and various contents of fatty acids. Gb3 data from our symptomatic *Gla^tm^Tg(CAG-A4GALT)* mice will give us insights into the organ-specific synthesis of ceramide.

**Table 4:** **Gb3 content and the Gb3 distributions of organs from *Gla^tm^Tg(CAG-A4GALT)* mice determined by deacylation and direct assays** Results are the mean ± S.D. of organ homogenates from four male mice (20 weeks old). The *p* values were calculated between deacylation and direct assay groups by a Student's *t* test. *n.s.*, not significant *versus* deacylation assay group. *, *p* < 0.05.

	Total Gb3	Gb3 isoform	Gb3(−2) analog	Gb3(+18) analog
	μ*g/mg protein*	% *of total content*		
**Deacylation assay**				
Heart	84.5 ± 21.8	89.6 ± 0.5	7.9 ± 0.7	2.2 ± 0.3
Kidney	117.1 ± 11.8	74.1 ± 1.3	5.1 ± 0.7	20.7 ± 2.0
Spleen	175.1 ± 24.4	97.3 ± 0.3	1.3 ± 0.2	1.3 ± 0.2
Liver	184.9 ± 33.0	83.9 ± 1.3	2.5 ± 0.3	13.4 ± 1.1
**Direct assay**				
Heart	88.4 ± 15.6 (n.s.)	87.7 ± 1.0*	8.6 ± 0.6 (n.s.)	3.7 ± 0.3*
Kidney	91.2 ± 12.3*	61.5 ± 1.0*	4.4 ± 0.8 (n.s.)	34.1 ± 1.1*
Spleen	138.0 ± 12.9*	96.2 ± 0.2*	1.8 ± 0.2 (n.s.)	2.0 ± 0.1*
Liver	153.8 ± 24.4 (n.s.)	78.3 ± 0.4*	2.6 ± 0.3 (n.s.)	19.1 ± 0.5*

### Limitations

Other types of ceramidases were not tested; therefore, it was not possible to establish the most effective enzyme. However, SCDase readily deacylated Gb3. Although the SCDase deacylation method was not directly compared with another method using a microwave (36), the enzymatic deacylation data in this study are reproducible.

### Conclusions

Two assays were established to determine total Gb3 concentrations by UPLC-MS/MS. These methods are useful not only for measuring total Gb3 concentration but also to determine the distribution of Gb3 analogs in different organs of symptomatic *Gla^tm^Tg(CAG-A4GALT)* mice. The Gb3 deacylation assay with SCDase is simple and reproducible and may show total Gb3 concentration more precisely. However, information regarding the fatty acid moiety of Gb3 could not be obtained. A method was also established for 37 Gb3 isoforms and analogs/isoforms via the synthesis of Gb3 molecules from lyso-Gb3 or its analogs and various fatty acids. Synthesized Gb3 molecules were useful for the determination of their cross-contamination on mass data, so that it was possible to establish the specific assay conditions in UPLC-MS/MS. We succeeded in the individual determination of structural isomers and could describe the percentage of Gb3(−2) analog/isoforms. Organ-specific Gb3 distribution was observed in the symptomatic *Gla^tm^Tg(CAG-A4GALT)* mice with progressive renal impairment. Total Gb3 contents in the spleen and liver were higher than that in the kidneys, but no abnormality in those organs was detected in Fabry model mice as well as Fabry patients. More than 25% of Gb3 were Gb3 analogs in the kidneys; the distribution of Gb3 analogs might relate to the organ dysfunction. The precise measurement of Gb3 content and analog proportions may allow more in-depth understanding of the pathophysiology of Fabry disease.

## Experimental procedures

### Nomenclature of Gb3 structure

Gb3 isoforms containing lyso-Gb3 moiety are expressed as Gb3(d18:1)(C*x*:*y*-OH), where the second paraenesis shows the structure of the fatty acid moiety, *x* and *y* indicate the number of carbons and their double bond, respectively, and -OH represents hydroxyl fatty acids. Gb3 analog Gb3(−2) containing lyso-Gb3(−2) is shown as Gb3(d18:2)(C*x*:*y*-OH), and Gb3 analog Gb3(+18) containing lyso-Gb3(+18) is shown as Gb3(d18:0-OH)(C*x*:*y*-OH).

### Chemicals and reagents

Standard Gb3 (porcine RBC), 2-hydroxydocosanoic acid (C22:0-OH), and 2-hydroxytetracosanoic acid (C24:0-OH) were purchased from Matreya LLC. Palmitic acid (C16:0), palmitoleic acid (C16:1), heptadecanoic acid (C17:0), stearic acid (C18:0), oleic acid (C18:1), linoleic acid (C18:2), arachidic acid (C20:0), cis-11-eicosenoic acid (C20:1), behenic acid (C22:0), erucic acid (C22:1), lignoceric acid (C24:0), and nervonic acid (C24:1) were obtained from Sigma–Aldrich. HPLC-grade methanol (MeOH), distilled water (H_2_O), acetonitrile (ACN), isopropyl alcohol (IPA), formic acid (FA), analytical grade chloroform (CHCl_3_), hexane, and other reagents were obtained from Fujifilm Wako Pure Chemical Corp. (Osaka, Japan). Iatrobeads were obtained from Iatron Laboratories (Tokyo, Japan).

### Purification of mouse organ Gb3

The symptomatic Fabry mouse model line *Gla^tm^Tg(CAG-A4GALT)* was generated by cross-breeding C57BL/6J-*Tg(CAG-A4GALT)* mice (30) and homozygous *Gla* knockout C57BL/6J;129S4-*Gla^tm1kul^* mice (4). All mice were housed under standard laboratory conditions in the animal facility of Niigata University and genotyped as described previously (6). The studies were conducted according to the principles and procedures outlined in the Science Council of Japan's Guidelines for Proper Conduct of Animal Experiments and were approved by the Presidents of Niigata University (SA00386 and SD01036). Approximately 5 g of each organ from 20-week-old *Gla^tm^Tg(CAG-A4GALT)* mice was collected and transferred to Oita University for Gb3 extraction. The organs were homogenized in H_2_O, glycosphingolipids were isolated and purified by solvent extraction and mild alkaline treatment, and Gb3 was purified by Iatrobeads column chromatography, as described previously (23).

### Treatment of purified Gb3 with SCDase

Aliquots (40 μl) of purified Gb3 (10 μg/ml) were mixed with 0.8 ml of CHCl_3_/MeOH (2:1 (v/v)) for lipid extraction, and aqueous lipids were separated by the addition of 0.2 ml of 1.5% FA in H_2_O and centrifugation at 6500 × *g* using a personal centrifuge (Gyrogen, Seoul, Korea) for 5 min. After removal of the upper layer, 40 μl of the lower layer was transferred to another tube and dried. Samples were suspended in 40 μl of enzyme mixture (5 μg/ml of SCDase in 25 mm sodium acetate buffer, pH 5.5, containing 0.5% Triton X-100, 5 mm CaCl_2_, and 5% DMSO) and incubated at 37 °C for 1 h. The reaction was stopped by the addition of 0.8 ml of CHCl_3_/MeOH (2:1 (v/v)). The reaction products were mixed with 20 μl of 100 ng/ml glycine derivative of lyso-Gb3 (Gly-lyso-Gb3 as an internal standard (37)) and 0.2 ml of 1.5% FA in H_2_O and extracted by centrifugation at 6500 × *g* for 5 min. Then 150 μl of the clear upper layer was transferred to another tube and dried using a centrifugal evaporator CVE-2000 (EYELA, Tokyo, Japan). Samples were redissolved in 50 μl of H_2_O and extracted with the same volume of H_2_O-saturated 1-butanol. The extracts were diluted with MeOH and analyzed by UPLC-MS/MS at GlycoPharma Corp.

### UPLC-MS/MS analysis for lyso-Gb3 and its analogs

Deacylated products (lyso-Gb3 and its eight analogs) were detected using a Xevo TQD MS with an Acquity UPLC system (Waters, Milford, MA), as described previously (27). All MS parameters were optimized to achieve the highest sensitivity for lyso-Gb3 analogs. Liquid chromatography and MS parameters are summarized in Table S1. The peak areas corresponding to lyso-Gb3 and its analogs in the multiple-reaction-monitoring (MRM) chromatogram were calculated using MassLynx software (Waters).

### Purification of lyso-Gb3, lyso-Gb3(−2), and lyso-Gb3(+18)

Approximately 1 mg of Gb3 purified from mouse kidneys was digested with 5 μg/ml SCDase at 37 °C for 1 h. The enzyme reaction was stopped by the addition of a 20-fold volume of CHCl_3_/MeOH (2:1 (v/v)). The products were extracted by the addition of a 5-fold volume of 1.5% FA in H_2_O, followed by centrifugation at 6500 × *g* for 5 min. The clear upper layer was transferred to another tube and mixed with a one-tenth volume of 1 n HCl. Each acidified sample was loaded on an Oasis MCX cartridge (500 mg, 60-μm particle size; Waters, Milford, MA) preconditioned with 6 ml of MeOH followed by 6 ml of 2% H_3_PO_4_ in H_2_O. After loading, the cartridge was washed with 3 ml of 2% FA in H_2_O, followed by 6 ml of 0.2% FA in MeOH. Lyso-Gb3 and its analogs were eluted with 2 ml of 3% ammonia in MeOH. After evaporation to dryness under a nitrogen stream, residues were reconstituted with 0.2 ml of MeOH, and 20 μl of sample was injected into the HPLC. Purification of lyso-Gb3 and its analogs was performed by HPLC (Jasco Co., Tokyo, Japan) with a SunFire C8 column (4.6 × 100 mm, 3.5-μm particle size; Waters). The flow rate was 0.7 ml/min, and the column temperature was 50 °C. Mobile phase A was ACN/IPA (60:40 (v/v)) + 0.2% FA, and mobile phase B was H_2_O/ACN (95:5 (v/v)) + 0.2% FA. The following gradient profile was used for purification: 0–8 min, 35–100% mobile phase A; 8–11 min, 100% A; 11–11.5 min, 100 to 35% A; and 11.5–16 min, 35% A. Lyso-Gb3(−2), lyso-Gb3, and lyso-Gb3(+18) were collected from 3.5–4.5, 6.5–7.0, and 7.0–7.5 min, respectively. The concentrations of final lyso-Gb3 preparations were determined by derivatization with *o*-phthaldialdehyde, as described previously (38).

### Synthesis of Gb3 molecules with SCDase

Individual Gb3 molecules were synthesized by the reverse reaction of SCDase with purified lyso-Gb3 or its analogs and purchased fatty acids. The preparations LG3, LG3(−2), and LG3(+18) described in Fig. 3 were used as purified lyso-Gb3, lyso-Gb3(−2), and lyso-Gb3(+18), respectively. For example, when a Gb3 isoform Gb3(d18:1)(C18:0) was synthesized, LG3 and stearic acid was used. Approximately 0.5 μg of LG3 was mixed with 25 μg of stearic acid dissolved in CHCl_3_/MeOH (2:1 (v/v)) and dried. Lipids were suspended in 40 μl of enzyme mixture (5 μg/ml of SCDase in 25 mm sodium acetate buffer (pH 5.5) containing 0.5% Triton X-100, 5 mm CaCl_2_, and 5% DMSO) and incubated at 37 °C for 1.5 h. The reaction was stopped by the addition of 0.8 ml of CHCl_3_/MeOH (2:1 (v/v)), and aqueous lipids were separated by the addition of 0.2 ml of 1.5% FA in H_2_O and centrifugation at 6500 × *g* for 5 min. After removal of the upper layer, the product was recovered in the lower layer and designated as LG3/C18:0.

In a manner similar to Gb3 isoforms, when Gb3 analogs Gb3(−2) and Gb3(+18) containing stearic acid were synthesized, LG3(−2) and LG3(+18) were used, respectively. All synthesized Gb3 standards were adjusted to concentrations at ∼3,000 peak area when determined by UPLC-MS/MS. In the direct assay, Gb3(d18:2)(C17:0) was used as an internal standard, which was synthesized enzymatically from LG3(−2) and heptadecanoic acid by the same method as described above.

### UPLC-MS/MS conditions for Gb3 direct assay

Gb3 isoforms and analogs were determined using a Xevo TQD mass spectrometer with an Acquity UPLC system (Waters). An Acquity UPLC BEH C18 column (2.1 × 100 mm, 1.7-μm particle size; Waters) was used for the chromatographic separation of Gb3 isoforms/analogs. The flow rate was 0.5 ml/min, and the column temperature was 60 °C using mobile phase A (H_2_O-ACN (95:5) + 0.1% FA) and mobile phase B (ACN-IPA-MeOH (40:40:20) + 0.1% FA). The column was equilibrated with 30% mobile phase A and 70% mobile phase B. The elution was started with a linear gradient from 70 to 100% mobile phase B in 1 min, followed by 2 min of using 100% mobile phase B, and then an immediate return to initial conditions for 2 min. MS/MS methods were set as Table S1. Retention time on the LC and basic mass condition for the detection of each Gb3 isoform and analog are summarized in Table 3. The cone voltage and collision energy were set as 120 and 60 V, respectively, for Gb3 in basic mass conditions. The peak areas corresponding to the Gb3 isoforms and analog/isoforms and the internal standard in the MRM chromatogram were calculated using MassLynx software (Waters).

### Gb3 deacylation and direct assays of homogenates from mouse organs

For the deacylation assay of homogenates from mouse organs, aliquots (40 μl) of organ homogenates (adjusted protein concentration: 1 mg/ml) were subjected to liquid extraction and SCDase treatment as described for purified Gb3. Deacylated products were determined by UPLC-MS/MS as described above.

For the direct assay, aliquots (20 μl) of tissue homogenates (1 mg/ml) were mixed with 0.4 ml of CHCl_3_/MeOH (2:1 (v/v)) for lipid extraction. After the addition of 20 μl of internal standard Gb3(d18:2)(C17:0) (40 ng/ml), aqueous lipids were separated by the addition of 0.1 ml of 1.5% FA in H_2_O and centrifugation at 6500 × *g* for 5 min. After removal of the upper layer, 40 μl of the lower layer was transferred to another tube and dried under a stream of nitrogen. Dried samples were subjected to mild alkaline treatment with 200 μl of 0.2 n NaOH in MeOH at 40 °C for 1 h. After neutralizing the solution with glacial acetic acid, 400 μl of CHCl_3_ and 150 μl of H_2_O were added to the sample solution, and the glycolipids were extracted by centrifugation at 6500 × *g* for 5 min. After removal of the upper layer, 160 μl of MeOH and 300 μl of hexane were added into the lower layer. Samples were applied to an Iatrobeads column (0.5 × 1 cm) equilibrated with hexane. After the column was washed with 0.6 ml of IPA-hexane (50:50 (v/v)), the bound glycolipids were eluted with 0.6 ml of IPA-hexane-H_2_O (50:35:15 (v/v/v)). Eluates were pooled, dried, and then dissolved with 0.1 ml of MeOH and analyzed by UPLC-MS/MS.

### Statistical analysis

The Shapiro–Wilk test was used to test for the normal distribution of variables. Data of each group were normally distributed and assessed for variance using the *F* test. All established two unpaired groups for comparisons showed homogeneous variances. A Student's *t* test was used for comparisons between two unpaired groups. Statistical analysis was performed using JMP®12 (SAS Institute, Cary, NC).

### Data availability

All of the data are contained within the article and supporting information.

## Author contributions

S. I., N. O., and H. M. conceptualization; S. I. and A. T. data curation; S. I. and A. T. formal analysis; S. I., M. I., and H. M. supervision; S. I. and A. T. investigation; S. I. and A. T. visualization; S. I., N. O., M. I., and H. M. methodology; S. I. writing-original draft; S. I. and H. M. project administration; A. T., N. O., M. I., and H. M. resources; A. T. software; A. T. and N. O. validation; A. T., N. O., M. I., and H. M. writing-review and editing; H. M. funding acquisition.

## Supplementary Material

Supporting Information

## Footnotes

2 The abbreviations used are:

Gb3: globotriaosylceramide
*Gla*: mouse α-galactosidase A gene
A4GALT: human α1 4-galactosyltransferase
ERT: enzyme replacement therapy
lyso-Gb3: globotriaosylsphingosine
SCDase: sphingolipid ceramide *N*-deacylase
UPLC: ultra-performance liquid chromatography
ACN: acetonitrile
IPA: isopropyl alcohol
FA: formic acid
Gly-lyso-Gb3: glycine derivative of lyso-Gb3
MRM: multiple-reaction-monitoring.
