# Supplementary material for: Determination of globotriaosylceramide analogs in the organs of a mouse model of Fabry disease
Source: J Biol Chem. 2020 Mar 16;295(17):5577–87. doi: 10.1074/jbc.RA120.012665 (PMC7186183; doi:10.1074/jbc.RA120.012665)
Supplement: Supporting Information [file supp_295_17_5577__index.html]

Determination of globotriaosylceramide analogs in the organs of a mouse model of Fabry disease — Determination of Gb3 analogs — Determination of globotriaosylceramide analogs in the organs of a mouse model of Fabry disease — Determination of Gb3 analogs — Supporting Information 

# Determination of globotriaosylceramide analogs in the organs of a mouse model of Fabry disease

## Supporting Information

- Supporting Information (to be published online) - Supporting Figures and Table
