# Supplementary material for: Determination of globotriaosylceramide analogs in the organs of a mouse model of Fabry disease
Source: J Biol Chem. 2020 Mar 16;295(17):5577–87. doi: 10.1074/jbc.RA120.012665 (PMC7186183; doi:10.1074/jbc.RA120.012665)
Supplement: Supporting Information [file supp_RA120.012665_158254_2_supp_491257_q70t96.pdf]

Supporting Information
for

Determination of globotriaosylceramide analogs in the organs of a mouse model of Fabry disease

Satoshi Ishii[1,2*], Atsumi Taguchi[3], Nozomu Okino[4], Makoto Ito[4], and Hiroki Maruyama[3]

[1]Department of Matrix Medicine, Faculty of Medicine, Oita University, Oita, Japan, [2]Biochemical Laboratory, GlycoPharma Corporation, Oita, Japan, [3]Department of Clinical Nephroscience, Niigata University Graduate School of Medical and Dental Sciences, Niigata, Japan, [4]Department of Bioscience and Biotechnology, Graduate School of Bioresource and Bioenvironmental Sciences, Kyushu University, Fukuoka, Japan.

Table of content

1. SUPPLEMENTARY FIGURES

Supplementary Figure S1. Effect of Gb3 digestion on the amount of SCDase (A), incubation time (B), and Gb3 content (C)

Supplementary Figure S2. TLC of Gb3 preparations extracted from the organs of *Gla^{tm}Tg(CAG-A4GALT)* mice

Supplementary Figure S3. Mass spectra of precursor and target ions

Supplementary Figure S4. Mass detection of synthesized Gb3 isoforms and analogs with C16 fatty acids

Supplementary Figure S5. Mass detection of synthesized Gb3 isoforms and analogs with C20 fatty acids

Supplementary Figure S6. Mass detection of synthesized Gb3 isoforms and analogs with C22 fatty acids

Supplementary Figure S7. Mass detection of synthesized Gb3 isoforms and analogs with C24 fatty acids

Supplementary Figure S8. UPLC–MS/MS chromatograms of the mixed Gb3 preparations containing C16 fatty acids

Supplementary Figure S9. UPLC–MS/MS chromatograms of the mixed Gb3 preparations containing C20 fatty acids

Supplementary Figure S10. UPLC–MS/MS chromatograms of the mixed Gb3 preparations containing C22 fatty acids

Supplementary Figure S11. UPLC–MS/MS chromatograms of the mixed Gb3 preparations containing C24 fatty acids

Supplementary Figure S12. Individual detection of structural isomers of Gb3

Supplementary Figure S13. Mass detection of synthesized Gb3 isoforms and analogs with hydroxy fatty acids

Supplementary Figure S14. UPLC–MS/MS chromatograms of the mixed Gb3 preparations containing hydroxy fatty acids

Supplementary Figure S15. MS data observed as peak area of individual or mixed Gb3 isoforms and analog/isoforms

2. SUPPLEMENTARY TABLE

Supplementary Table S1. UPLC–MS/MS parameters used for the determination of lyso-Gb3 and its analogs

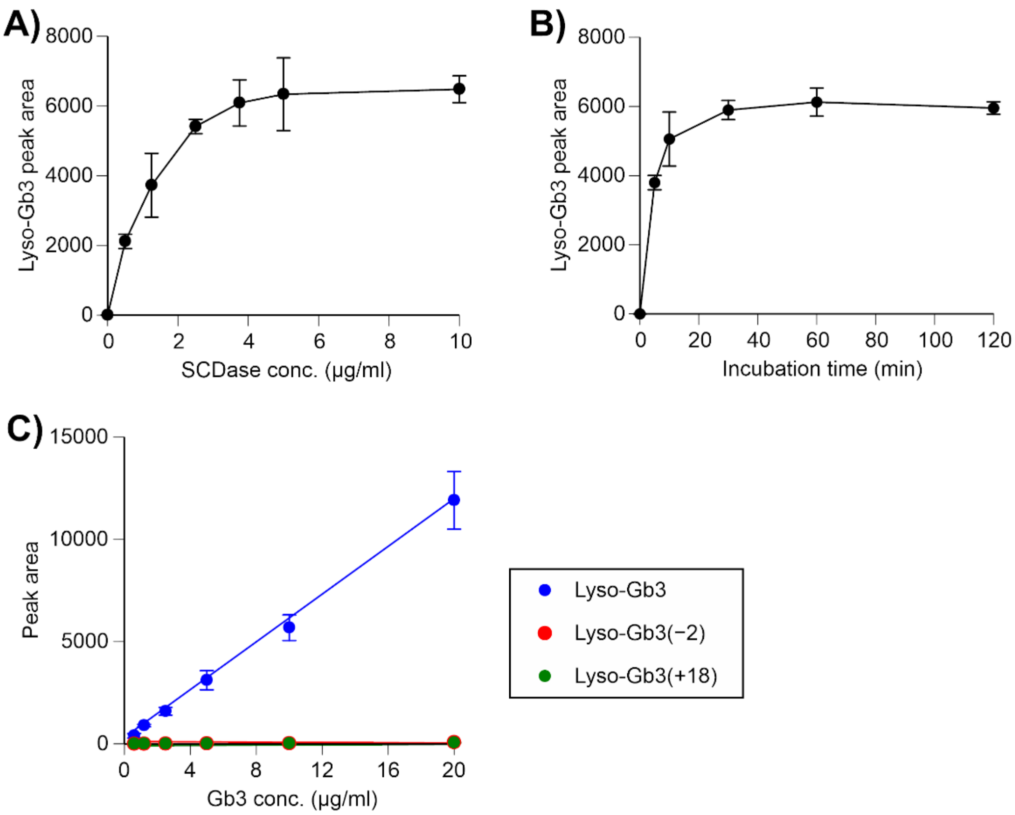

Supplementary Figure S1. Effect of Gb3 digestion on the amount of SCDase (A), incubation time (B), and Gb3 content (C). Standard Gb3 (10 μg/ml) was treated with the indicated amounts of SCDase for 1 h in A, and digested with 5 μg/mL SCDase for the indicated periods in B. In C, standard Gb3 solutions (0.6–20 μg/ml) were incubated with 5 μg/ml SCDase for 1 h. Results are the mean ± standard deviation (SD) of four independent assays.

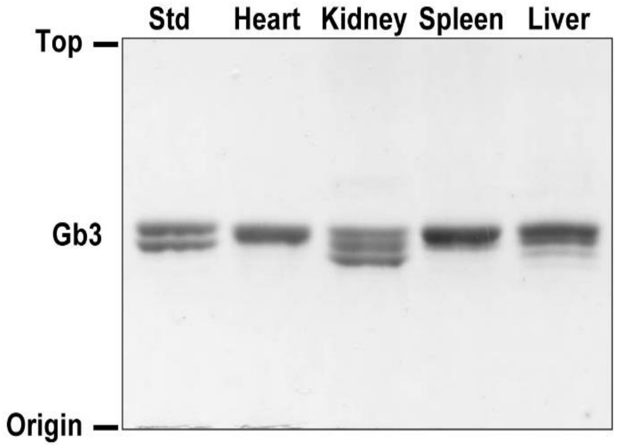

Supplementary Figure S2. TLC of Gb3 preparations extracted from the organs of *Gla^{tm}Tg(CAG-A4GALT)* mice. Final preparations of purified Gb3 from various organs were subjected to TLC and visualized with orcinol-sulfuric acid reagent. Std, standard Gb3.

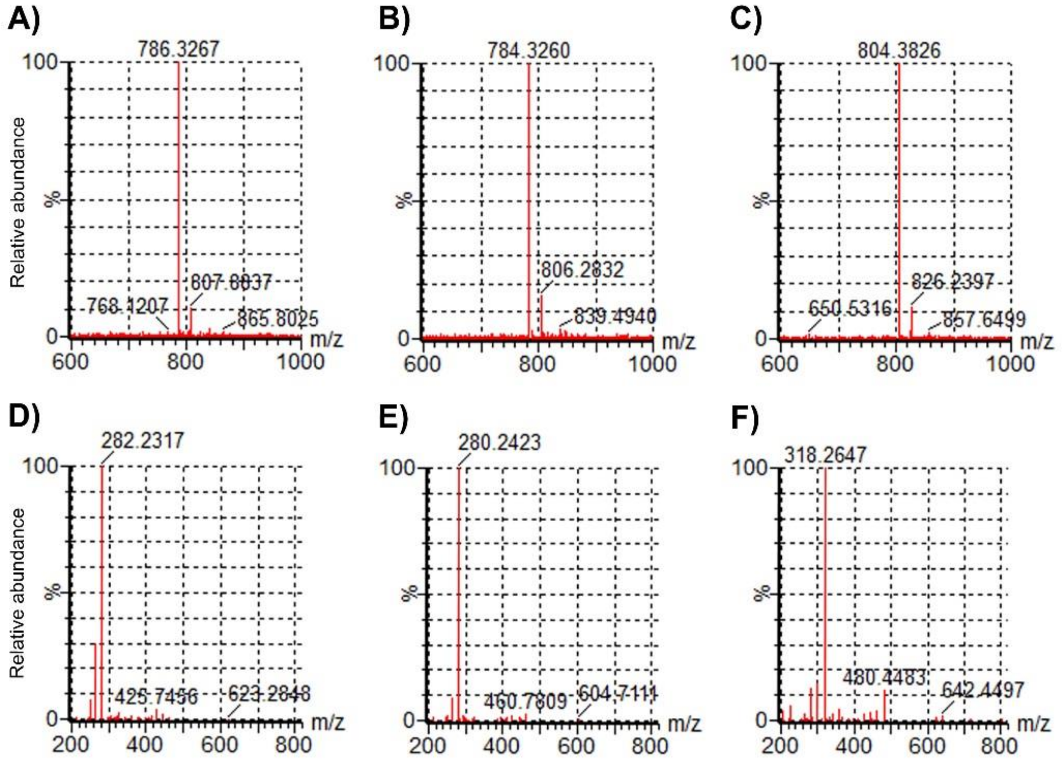

Supplementary Figure S3. Mass spectra of precursor and target ions. Precursor ions in the final LG3 (A), LG3(−2) (B), and LG3(+18) (C) preparations were determined in multiscan mode. MS/MS fragmentation spectra of target ions in LG3 (D), LG3(−2) (E), and LG3(+18) (F) were determined using the precursor ion masses 786.3, 784.3, and 804.3 *m/z*, respectively. MS conditions are described in Table 1.

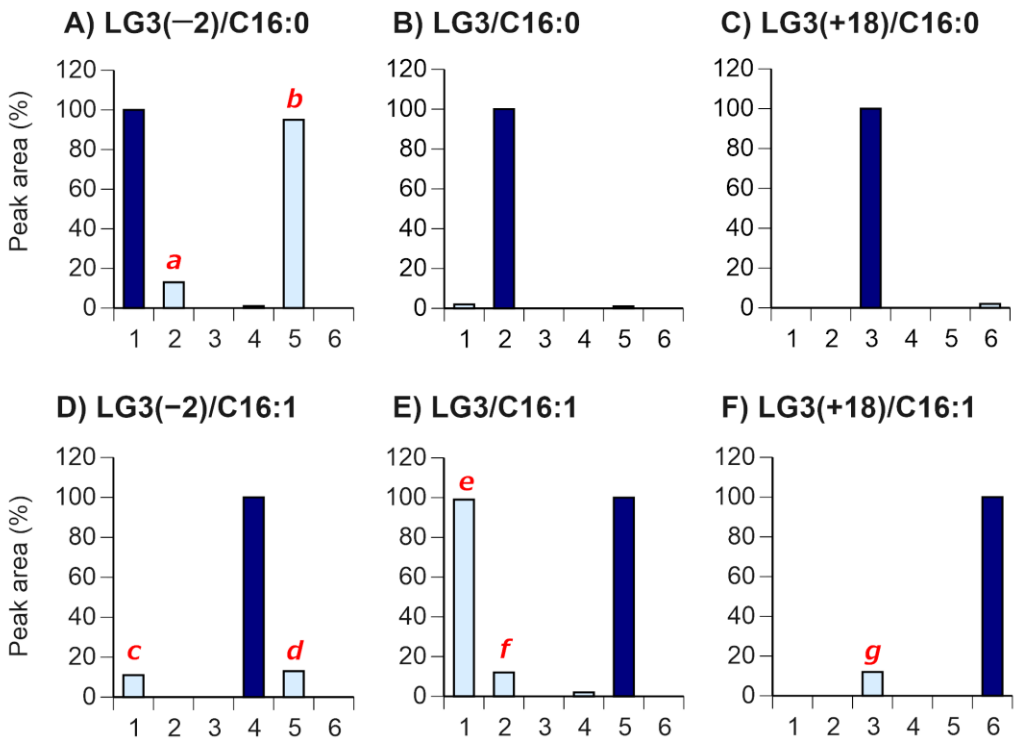

Supplementary Figure S4. Mass detection of synthesized Gb3 isoforms and analogs with C16 fatty acids. Synthesized Gb3 isoforms and analogs were applied to UPLC–MS/MS and six different transition parameters were determined at a narrow retention time range. Numbers along the x-axis are transition parameters; 1, 1044.7 > 882.6 for Gb3(d18:2)(C16:0); 2, 1046.7 > 884.6 for Gb3(d18:1)(C16:0); 3, 1064.7 > 902.6 for Gb3(d18:0-OH)(C16:0); 4, 1042.7 > 880.6 for Gb3(d18:2)(C16:1); 5, 1044.7 > 882.6 for Gb3(d18:1)(C16:1); 6, 1062.7 > 900.6 for Gb3(d18:0-OH)(C16:1). Retention times are 1.53 ± 0.04 min in (A), 1.61 ± 0.04 min in (B), 1.57 ± 0.04 min in (C), 1.42 ± 0.04 min in (D), 1.53 ± 0.04 min in (E) and 1.47 ± 0.04 min in (F), respectively. The authentic peak of the molecule (dark blue bar) was defined as 100%. Results are the mean of three independent assays.

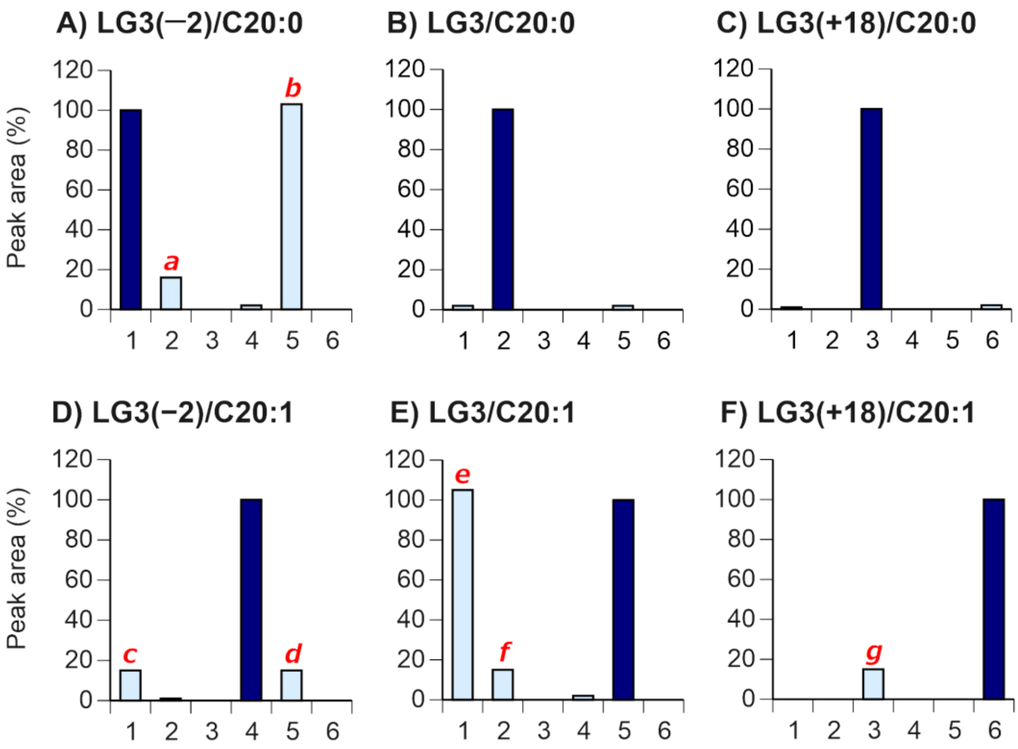

Supplementary Figure S5. Mass detection of synthesized Gb3 isoforms and analogs with C20 fatty acids. Synthesized Gb3 isoforms and analogs were applied to UPLC–MS/MS and six different transition parameters were determined at a narrow retention time range. Numbers along the x-axis are transition parameters; 1, 1100.7 > 938.6 for Gb3(d18:2)(C20:0); 2, 1102.7 > 940.6 for Gb3(d18:1)(C20:0); 3, 1120.7 > 958.6 for Gb3(d18:0-OH)(C20:0); 4, 1098.7 > 936.6 for Gb3(d18:2)(C20:1); 5, 1100.7 > 938.6 for Gb3(d18:1)(C20:1); 6, 1118.7 > 956.6 for Gb3(d18:0-OH)(C20:1). Retention times are 1.69 ± 0.04 min in (A), 1.77 ± 0.04 min in (B), 1.73 ± 0.04 min in (C), 1.62 ± 0.04 min in (D), 1.69 ± 0.04 min in (E) and 1.65 ± 0.04 min in (F), respectively. The authentic peak of the molecule (dark blue bar) was defined as 100%. Results are the mean of three independent assays.

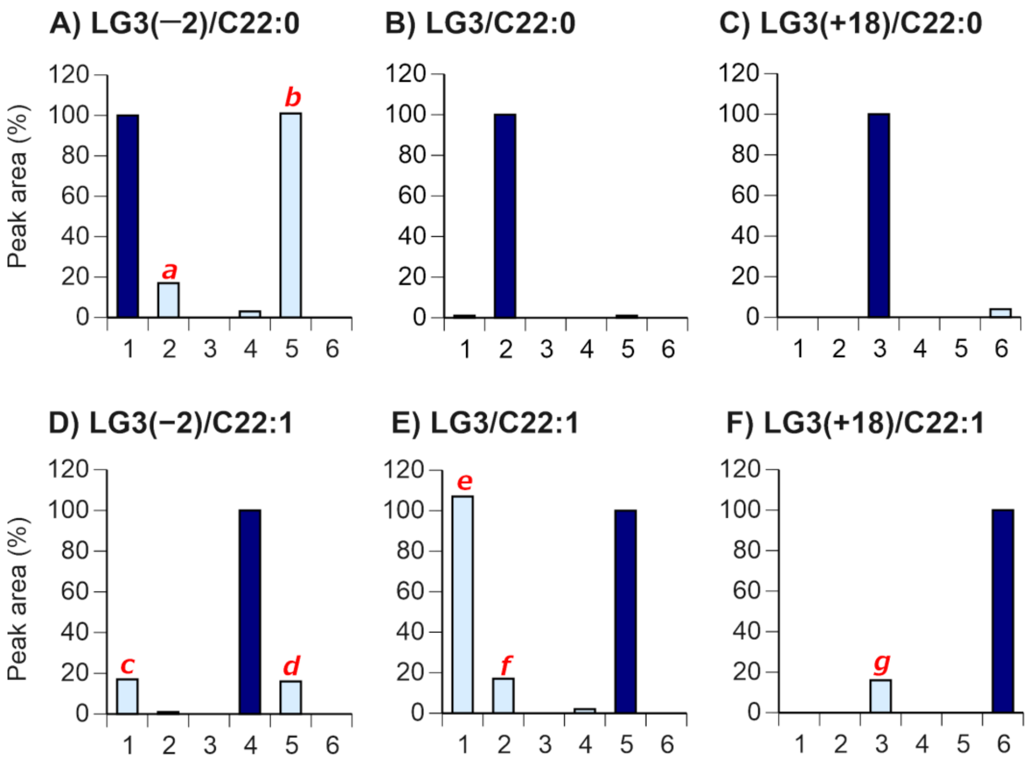

Supplementary Figure S6. Mass detection of synthesized Gb3 isoforms and analogs with C22 fatty acids. Synthesized Gb3 isoforms and analogs were applied to UPLC–MS/MS and six different transition parameters were determined at a narrow retention time range. Numbers along the x-axis are transition parameters; 1, 1128.7 > 966.6 for Gb3(d18:2)(C22:0); 2, 1130.7 > 968.6 for Gb3(d18:1)(C22:0); 3, 1148.7 > 986.6 for Gb3(d18:0-OH)(C22:0); 4, 1126.7 > 964.6 for Gb3(d18:2)(C22:1); 5, 1128.7 > 966.6 for Gb3(d18:1)(C22:1); 6, 1146.7 > 984.6 for Gb3(d18:0-OH)(C22:1). Retention times are 1.76 ± 0.04 min in (A), 1.84 ± 0.04 min in (B), 1.81 ± 0.04 min in (C), 1.66 ± 0.04 min in (D), 1.76 ± 0.04 min in (E) and 1.72 ± 0.04 min in (F), respectively. The authentic peak of the molecule (dark blue bar) was defined as 100%. Results are the mean of three independent assays. The authentic peak of the molecule (dark blue bar) was defined as 100%. Results are the mean of three independent assays.

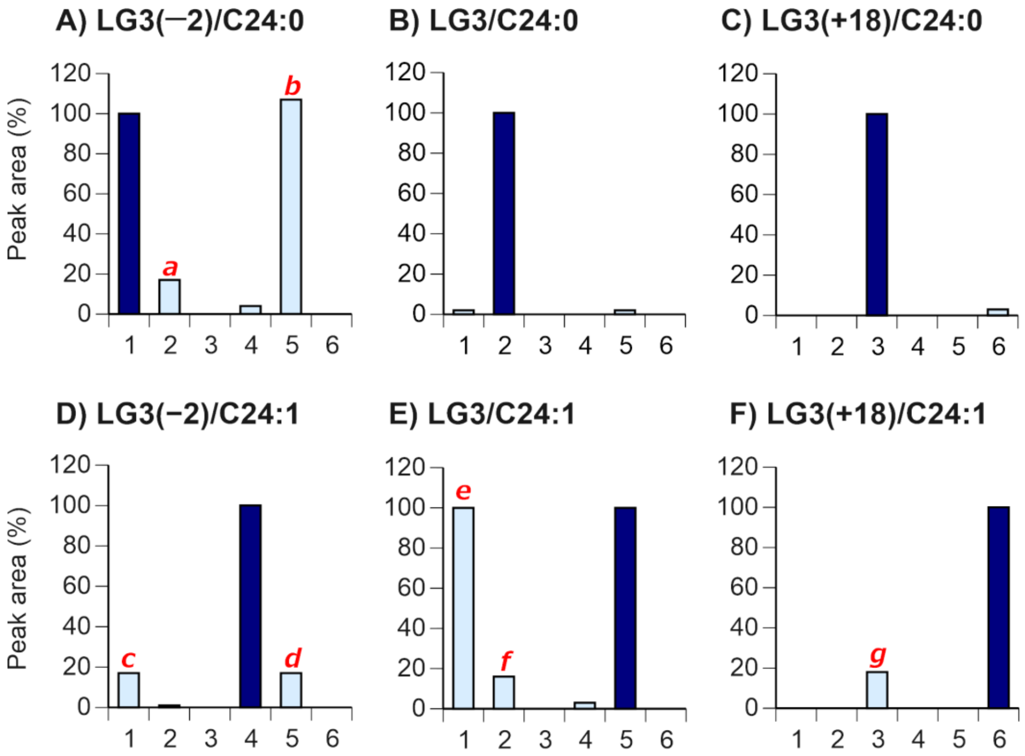

Supplementary Figure S7. Mass detection of synthesized Gb3 isoforms and analogs with C24 fatty acids. Synthesized Gb3 isoforms and analogs were applied to UPLC–MS/MS and six different transition parameters were determined at a narrow retention time range. Numbers along the x-axis are transition parameters; 1, 1156.7 > 994.6 for Gb3(d18:2)(C24:0); 2, 1158.7 > 996.6 for Gb3(d18:1)(C24:0); 3, 1176.7 > 1014.6 for Gb3(d18:0-OH)(C24:0); 4, 1154.7 > 992.6 for Gb3(d18:2)(C24:1); 5, 1156.7 > 994.6 for Gb3(d18:1)(C24:1); 6, 1174.7 > 1012.6 for Gb3(d18:0-OH)(C24:1). Retention times are 1.83 ± 0.04 min in (A), 1.91 ± 0.04 min in (B), 1.88 ± 0.04 min in (C), 1.75 ± 0.04 min in (D), 1.83 ± 0.04 min in (E) and 1.79 ± 0.04 min in (F), respectively. The authentic peak of the molecule (dark blue bar) was defined as 100%. Results are the mean of three independent assays.

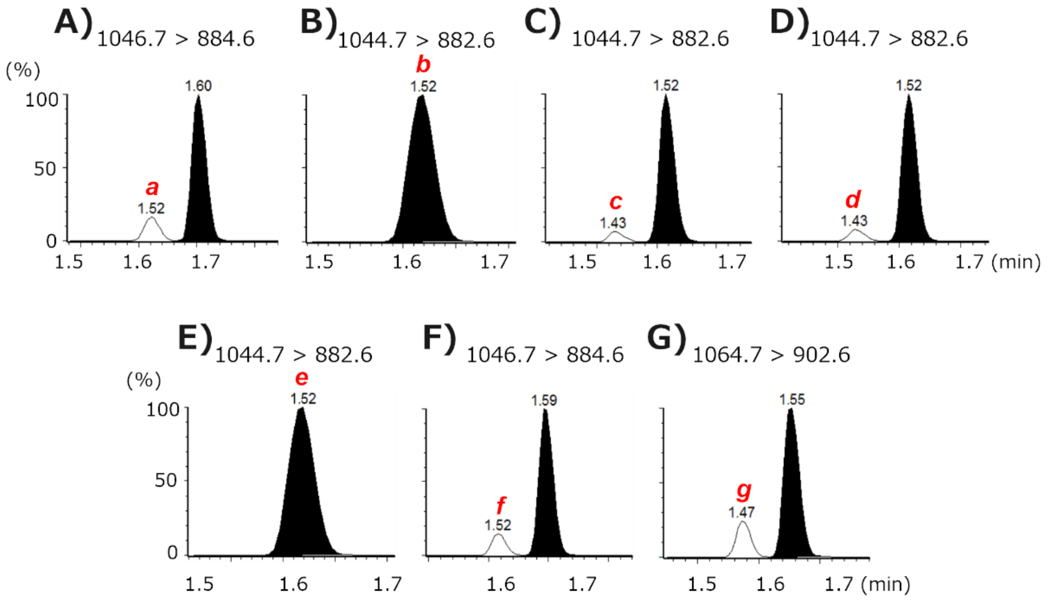

Supplementary Figure S8. UPLC–MS/MS chromatograms of the mixed Gb3 preparations containing C16 fatty acids. In (A), LG3(−2)/C16:0 and LG3/C16:0 were mixed and applied to UPLC–MS/MS, and detected with the transition parameter 2 in the legend to Fig. S4 (1046.7 > 884.6) at the retention time (1.61 ± 0.04 min). (B), LG3(−2)/C16:0 and LG3/C16:1 mixture, parameter 5 (1044.7 > 882.6), at (1.53 ± 0.04 min); (C), LG3(−2)/C16:1 and LG3(−2)/C16:0 mixture, parameter 1 (1044.7 > 882.6), at (1.53 ± 0.04 min); (D) LG3(−2)/C16:1 and LG3/C16:1 mixture, parameter 5 (1044.7 > 882.6), at (1.53 ± 0.04 min); (E) LG3/C16:1 and LG3(−2)/C16:0 mixture, parameter 1 (1044.7 > 882.6), at (1.53 ± 0.04 min); (F) LG3/C16:1 and LG3/C16:0 mixture, parameter 2 (1046.7 > 884.6), at (1.61 ± 0.04 min); (G) LG3(+18)/C16:1 and LG3(+18)/C16:0 mixture, parameter 3 (1064.7 > 902.6), at (1.57 ± 0.04 min).

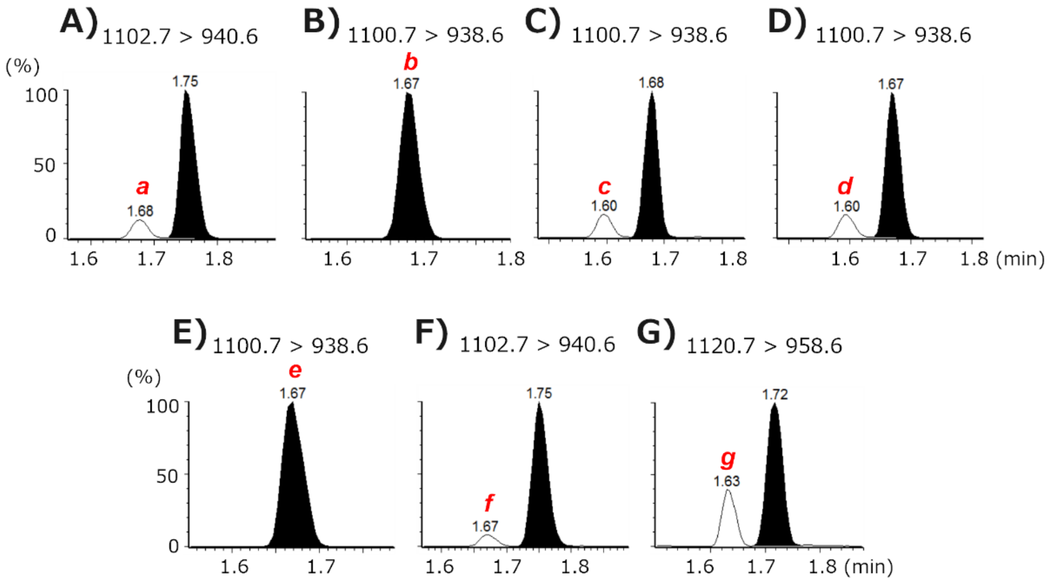

Supplementary Figure S9. UPLC–MS/MS chromatograms of the mixed Gb3 preparations containing C20 fatty acids. In (A), LG3(−2)/C20:0 and LG3/C20:0 were mixed and applied to UPLC–MS/MS, and detected with the transition parameter 2 in the legend to Fig. S5 (1102.7 > 940.6) at the retention time (1.77 ± 0.04 min). (B), LG3(−2)/C20:0 and LG3/C20:1 mixture, parameter 5 (1100.7 > 938.6), at (1.69 ± 0.04 min); (C), LG3(−2)/C20:1 and LG3(−2)/C20:0 mixture, parameter 1 (1100.7 > 938.6), at (1.69 ± 0.04 min); (D) LG3(−2)/C20:1 and LG3/C20:1 mixture, parameter 5 (1100.7 > 938.6), at (1.69 ± 0.04 min); (E) LG3/C20:1 and LG3(−2)/C20:0 mixture, parameter 1 (1100.7 > 938.6), at (1.69 ± 0.04 min); (F) LG3/C20:1 and LG3/C20:0 mixture, parameter 2 (1102.7 > 940.6), at (1.77 ± 0.04 min); (G) LG3(+18)/C20:1 and LG3(+18)/C20:0 mixture, parameter 3 (1120.7 > 958.6), at (1.73 ± 0.04 min).

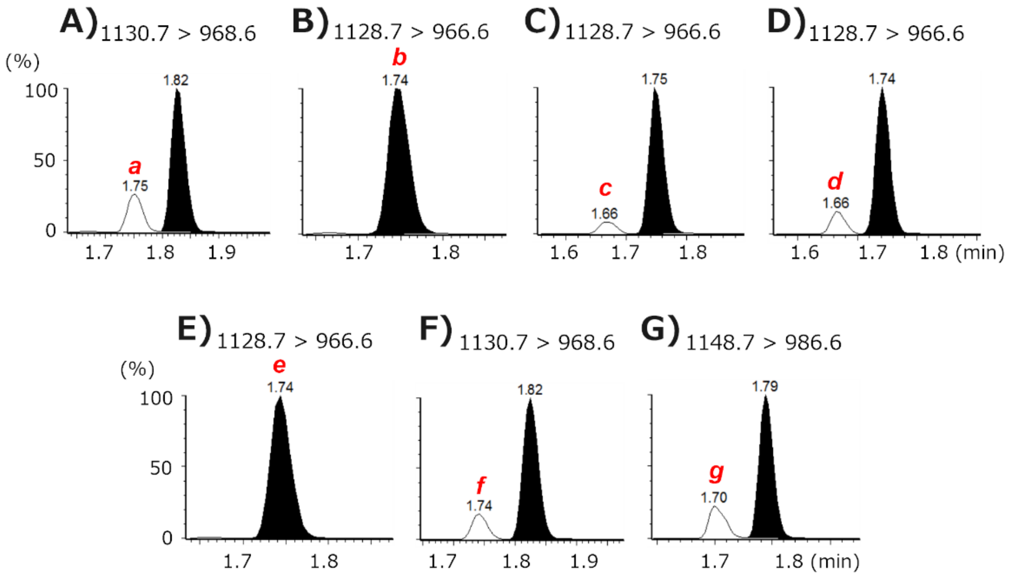

Supplementary Figure S10. UPLC–MS/MS chromatograms of the mixed Gb3 preparations containing C22 fatty acids. In (A), LG3(−2)/C22:0 and LG3/C22:0 were mixed and applied to UPLC–MS/MS, and detected with the transition parameter 2 in the legend to Fig. S6 (1130.7 > 968.6) at the retention time (1.84 ± 0.04 min). (B), LG3(−2)/C22:0 and LG3/C22:1 mixture, parameter 5 (1128.7 > 966.6), at (1.76 ± 0.04 min); (C), LG3(−2)/C22:1 and LG3(−2)/C22:0 mixture, parameter 1 (1128.7 > 966.6), at (1.76 ± 0.04 min); (D) LG3(−2)/C22:1 and LG3/C22:1 mixture, parameter 5 (1128.7 > 966.6), at (1.76 ± 0.04 min); (E) LG3/C22:1 and LG3(−2)/C22:0 mixture, parameter 1 (1128.7 > 966.6), at (1.76 ± 0.04 min); (F) LG3/C22:1 and LG3/C22:0 mixture, parameter 2 (1130.7 > 968.6), at (1.84 ± 0.04 min); (G) LG3(+18)/C22:1 and LG3(+18)/C22:0 mixture, parameter 3 (1148.7 > 986.6), at (1.81 ± 0.04 min).

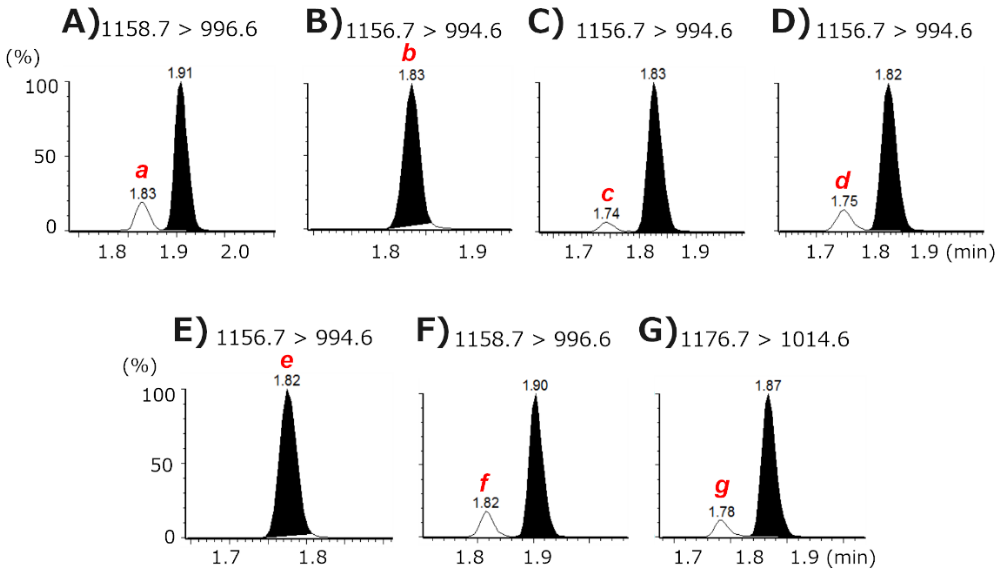

Supplementary Figure S11. UPLC–MS/MS chromatograms of the mixed Gb3 preparations containing C24 fatty acids. In (A), LG3(−2)/C24:0 and LG3/C24:0 were mixed and applied to UPLC–MS/MS, and detected with the transition parameter 2 in the legend to Fig. S7 (1158.7 > 996.6) at the retention time (1.91 ± 0.04 min). (B), LG3(−2)/C24:0 and LG3/C24:1 mixture, parameter 5 (1156.7 > 994.6), at (1.83 ± 0.04 min); (C), LG3(−2)/C24:1 and LG3(−2)/C24:0 mixture, parameter 1 (1156.7 > 994.6), at (1.83 ± 0.04 min); (D) LG3(−2)/C24:1 and LG3/C24:1 mixture, parameter 5 (1156.7 > 994.6), at (1.83 ± 0.04 min); (E) LG3/C24:1 and LG3(−2)/C24:0 mixture, parameter 1 (1156.7 > 994.6), at (1.83 ± 0.04 min); (F) LG3/C24:1 and LG3/C24:0 mixture, parameter 2 (1158.7 > 996.6), at (1.91 ± 0.04 min); (G) LG3(+18)/C24:1 and LG3(+18)/C24:0 mixture, parameter 3 (1176.7 > 1014.6), at (1.88 ± 0.04 min).

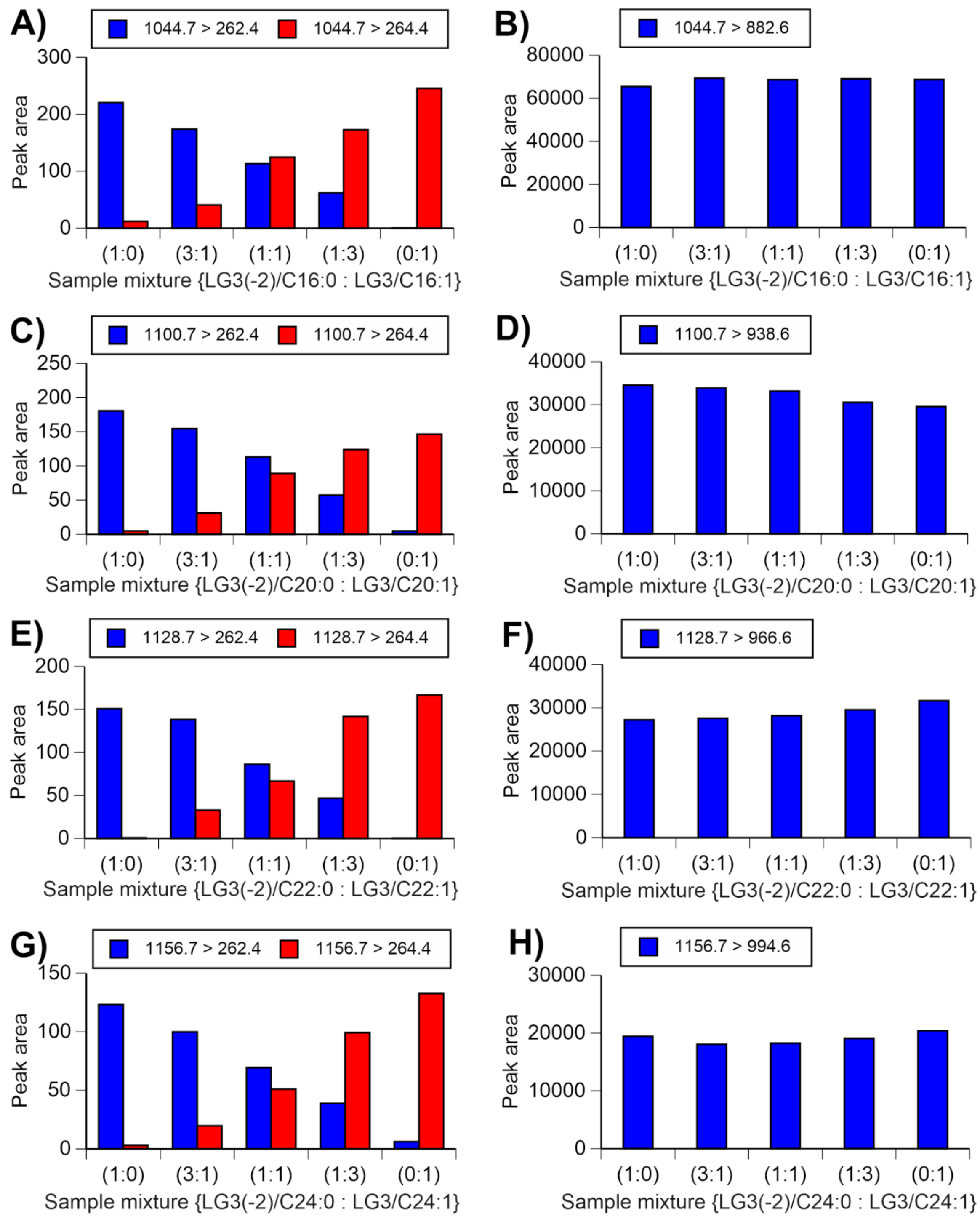

Supplementary Figure S12. Individual detection of structural isomers of Gb3. The mixtures of LG3(−2)/C16:0 and LG3/C16:1 at the indicated ratio were determined by the backbone mass conditions (A) or basic mass condition (B). The mixtures of LG3(−2)/C20:0 and LG3/C20:1, LG3(−2)/C22:0 and LG3/C22:1 and LG3(−2)/C24:0 and LG3/C24:1 were also subjected to the backbone assays (C), (E) and (G), respectively, and basic assays (D), (F) and (H), respectively.

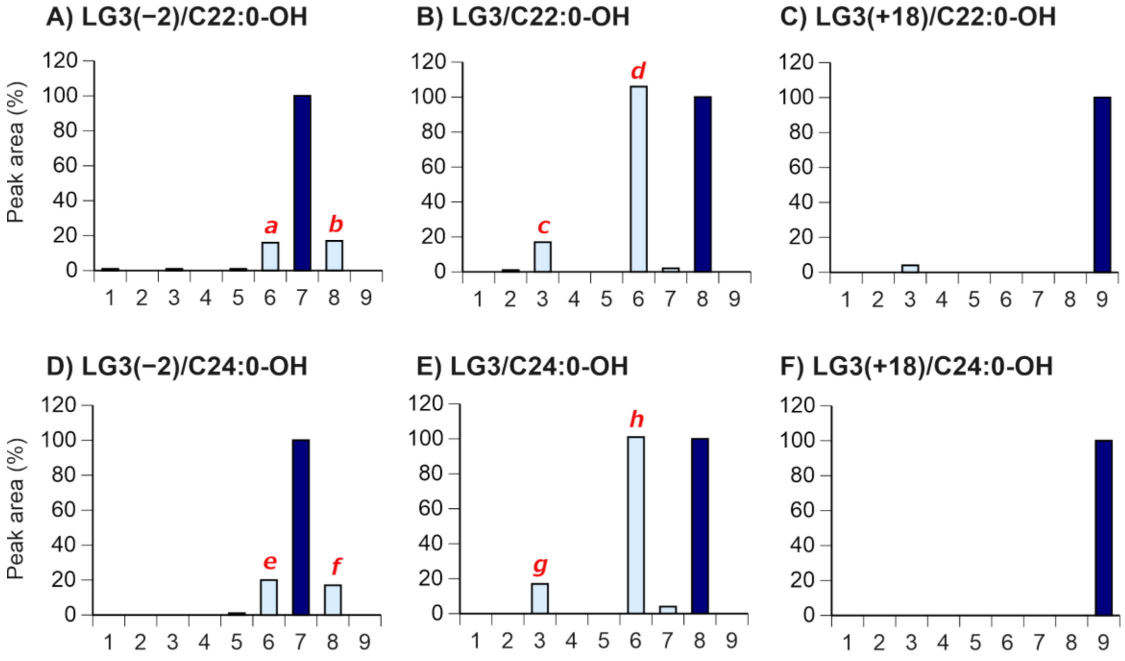

Supplementary Figure S13. Mass detection of synthesized Gb3 isoforms and analogs with hydroxy fatty acids. Each synthesized Gb3 preparation was applied to UPLC–MS/MS and nine different transition parameters were determined at a narrow retention time range. In A–C, numbers along the x-axis are transition parameters; 1, 1128.7 > 966.6 for Gb3(d18:2)(C22:0); 2, 1130.7 > 968.6 for Gb3(d18:1)(C22:0); 3, 1148.7 > 986.6 for Gb3(d18:0-OH)(C22:0); 4, 1126.7 > 964.6 for Gb3(d18:2)(C22:1); 5, 1128.7 > 966.6 for Gb3(d18:1)(C22:1); 6, 1146.7 > 984.6 for Gb3(d18:0-OH)(C22:1); 7, 1144.7 > 982.6 for Gb3(d18:2)(C22:0-OH); 8, 1146.7 > 984.6 for Gb3(d18:1)(C22:0-OH); and 9, 1164.7 > 1002.6 for Gb3(d18:0-OH)(C22:0-OH). Retention times are 1.75 ± 0.04 min in (A), 1.83 ± 0.04 min in (B), and 1.80 ± 0.04 min in (C), respectively. In D–F, numbers along the x-axis are transition parameters; 1, 1156.7 > 994.6 for Gb3(d18:2)(C24:0); 2, 1158.7 > 996.6 for Gb3(d18:1)(C24:0); 3, 1176.7 > 1014.6 for Gb3(d18:0-OH)(C24:0); 4, 1154.7 > 992.6 for Gb3(d18:2)(C24:1); 5, 1156.7 > 994.6 for Gb3(d18:1)(C24:1); 6, 1174.7 > 1012.6 for Gb3(d18:0-OH)(C24:1); 7, 1172.7 > 1010.6 for Gb3(d18:2)(C24:0-OH); 8, 1174.7 > 1012.6 for Gb3(d18:1)(C24:0-OH); and 9, 1192.7 > 1030.6 for Gb3(d18:0-OH)(C24:0-OH). Retention times are 1.81 ± 0.04 min in (D), 1.90 ± 0.04 min in (E) and 1.87 ± 0.04 min in (F), respectively. The authentic peak of the molecule (dark blue bar) was defined as 100%. Results are the mean of three independent assays.

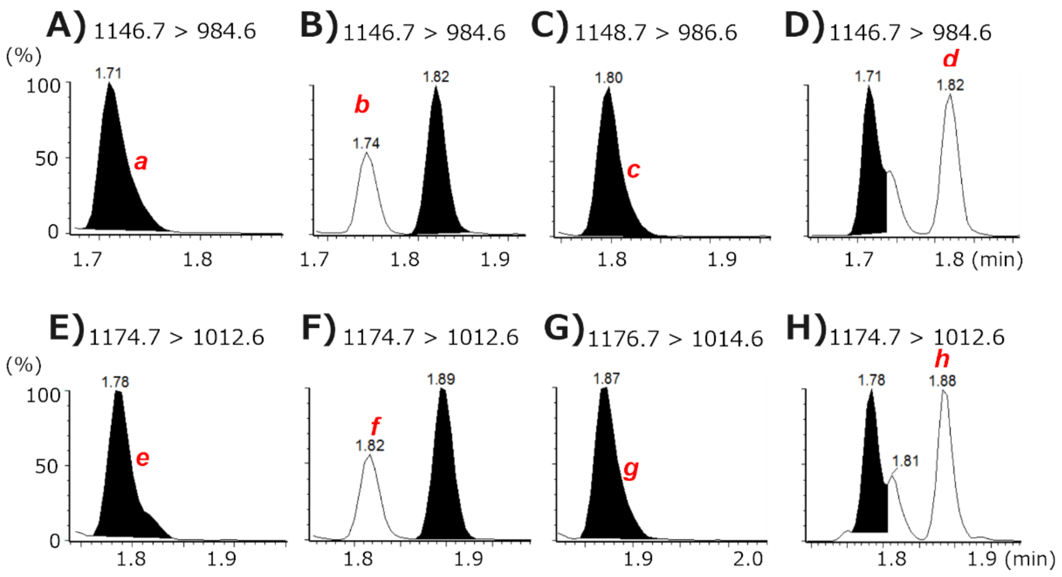

Supplementary Figure S14. UPLC–MS/MS chromatograms of the mixed Gb3 preparations containing hydroxy fatty acids. In (A), LG3(−2)/C22:0-OH and LG3(+18)/C22:1 were mixed and applied to UPLC–MS/MS, and detected with the transition parameter 6 in the legend to Fig. S13 (1146.7 > 984.6) at the retention time (1.72 ± 0.04 min). (B), LG3(−2)/C22:0-OH and LG3/C22:0-OH mixture, parameter 8 (1146.7 > 984.6), at (1.83 ± 0.04); (C), LG3/C22:0-OH and LG3(+18)/C22:0 mixture, parameter 3 (1148.7 > 986.6), at (1.81 ± 0.04); (D) LG3/C22:0-OH and LG3(+18)/C22:1 mixture, parameter 6 (1146.7 > 984.6), at (1.72 ± 0.04); (E) LG3(−2)/C24:0-OH and LG3(+18)/C24:1 mixture, parameter 6 (1174.7 > 1012.6), at (1.79 ± 0.04); (F) LG3(−2)/C24:0-OH and LG3/C24:0-OH mixture, parameter 8 (1174.7 > 1012.6), at (1.90 ± 0.04); (G) LG3/C24:0-OH and LG3(+18)/C24:0 mixture, parameter 3 (1176.7 > 1014.6), at (1.88 ± 0.04); and (H) LG3/C24:0-OH and LG3(+18)/C24:1 mixture, parameter 6 (1174.7 > 1012.6), at (1.79 ± 0.04).

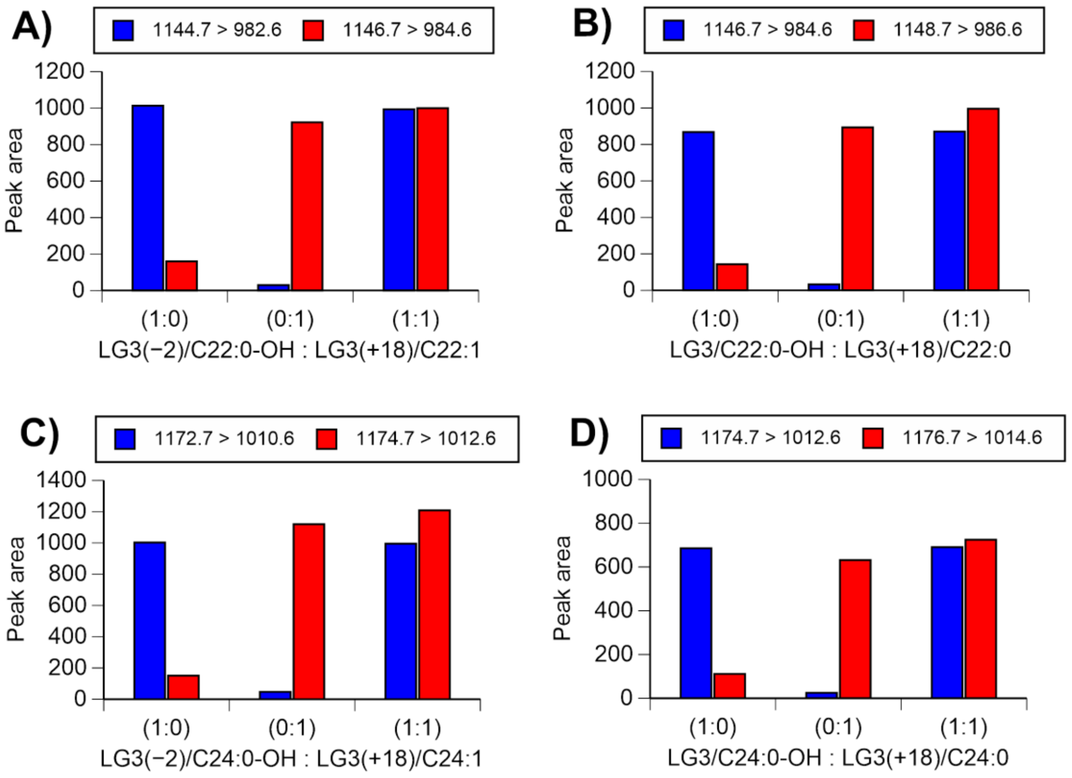

Supplementary Figure S15. MS data observed as peak area of individual or mixed Gb3 isoforms and analog/isoforms. The MS data of Gb3(d18:0-OH)(C22:1), Gb3(d18:0-OH)(C22:0) were slightly increased by the addition of Gb3(d18:2)(C22:0-OH), Gb3(d18:1)(C22:0-OH), respectively, in (A) and (B). Same effects of Gb3(d18:2)(C24:0-OH) and Gb3(d18:1)(C24:0-OH) were observed in (C) and (D), respectively.

Supplementary Table S1 UPLC–MS/MS parameters used for the determination of lyso-Gb3 and its analogs.

UPLC method		MS/MS method	
Column	UPLC HSS T3, 1.8 µm, 2.1 mm × 50 mm	Operating mode	MRM
Temperature	45°C	Ionization	ESI+
Mobile phase A	H_2O + 0.1% FA	Capillary voltage	3.5 kV
Mobile phase B	MeOH + 0.1% FA	Extraction cone voltage	55 V
Flow rate	0.6 ml/min	Desolvation temperature	300°C
**Gradient**		Desolvation gas flow	900 L/h
0–0.8 min	50–100% B	Cone gas flow	50 L/h
0.8–2.8 min	100% B	Collision energy MS	3 V
2.8–2.9 min	100–50% B		
2.9–5 min	50% B		

Compound	Acquisition parameters			
	**Transition**	**Dwell time (s)**	**Cone voltage (V)**	**Collision energy (V)**
Lyso-Gb3	786.3 > 282.3	0.03	60	35
Lyso-Gb3 (−28)	758.3 > 254.3	0.03	50	40
Lyso-Gb3 (−12)	774.3 > 252.3	0.03	45	40
Lyso-Gb3 (−2)	784.3 > 280.3	0.03	60	35
Lyso-Gb3 (+14)	800.3 > 278.3	0.03	60	35
Lyso-Gb3 (+16)	802.3 > 280.3	0.03	55	40
Lyso-Gb3 (+18)	804.3 > 318.3	0.03	60	35
Lyso-Gb3 (+34)	820.3 > 334.3	0.03	75	35
Lyso-Gb3 (+50)	836.3 > 350.3	0.03	70	35
Gly-lyso-Gb3	843.3 > 339.3	0.03	40	30
